# Unraveling Autonomic Dysfunction in GBA‐Related Parkinson's Disease

**DOI:** 10.1002/mdc3.13892

**Published:** 2023-10-13

**Authors:** Grazia Devigili, Giulia Straccia, Emanuele Cereda, Barbara Garavaglia, Alessandro Fedeli, Antonio Emanuele Elia, Sylvie Hélène Marie Jeanne Piacentini, Sara Prioni, Paolo Amami, Federica Invernizzi, Nico Golfrè Andreasi, Luigi Michele Romito, Roberto Eleopra, Roberto Cilia

**Affiliations:** ^1^ Fondazione IRCCS Istituto Neurologico Carlo Besta, Department of Clinical Neurosciences, Parkinson and Movement Disorders Unit Milan Italy; ^2^ Neurology and Stroke Unit C.T.O. Hospital, A.O.R.N Ospedali dei Colli Naples Italy; ^3^ Clinical Nutrition and Dietetics Unit Fondazione IRCCS Policlinico San Matteo Pavia Italy; ^4^ Fondazione IRCCS Istituto Neurologico Carlo Besta, Unit of Medical Genetics and Neurogenetics Milan Italy; ^5^ Neuropsychology Unit Fondazione IRCCS Istituto Neurologico Carlo Besta Milan Italy

**Keywords:** Parkinson's disease, GBA, autonomic dysfunction, spectral analysis, sudomotor dysfunction

## Abstract

**Background:**

Patients with Parkinson's disease (PD) and *GBA* gene mutations (GBA‐PD) develop nonmotor complications more frequently than noncarriers. However, an objective characterization of both cardiovascular and sudomotor autonomic dysfunction using extensive clinical and instrumental measures has never been provided so far. Survival is reduced in GBA‐PD regardless of age and dementia, suggesting that other hitherto unrecognized factors are involved.

**Objectives:**

To provide instrumental measures of pattern and severity of autonomic dysfunction in GBA‐PD and explore their correlation with other non‐motor symptoms and implications for clinical practice.

**Methods:**

In this cross‐sectional study, 21 GBA‐PD and 24 matched PD noncarriers underwent extensive assessment of motor and non‐motor features, including neuropsychological testing. Cardiovascular autonomic function was explored through a comprehensive battery of indexes, including power spectral analysis of the R‐R intervals and blood pressure short‐term variability during resting state and active maneuvers. Dynamic Sweat Test was used to assess post‐ganglionic sudomotor dysfunction.

**Results:**

Despite minimal or absent clinical correlates, cardiovagal and sympathetic indexes, heart rate variability parameters and sudomotor postganglionic function were more severely impaired in GBA‐PD than noncarriers (overcoming relatively preserved compensatory peripheral sympathetic function), suggesting more prominent cardiac sympatho‐vagal demodulation, efferent baroreflex failure and peripheral sympathetic dysfunction in GBA‐PD. Cardiovascular dysautonomia showed marginal correlations with cognitive impairment.

**Conclusions:**

Compared to PD noncarriers, GBA‐PD display more severe instrumental autonomic abnormalities, which may be underestimated by purely clinical measures, despite their relevance on morbidity and mortality. This supports the necessity of implementing instrumental autonomic assessment in all GBA‐PD, regardless of clinically overt symptoms.

Heterozygous variants in the *GBA* gene, encoding for the lysosomal enzyme Glucocerebrosidase (GCase), are the most frequent genetic risk factor for Parkinson's disease (PD).^1^, ^2^ They convey the strongest lifetime risk for PD^3^ and associate with faster motor and non‐motor decline, ultimately reducing survival.^4^, ^5^, ^6^, ^7^, ^8^ The increased mortality risk in PD carriers of *GBA* mutations (GBA‐PD) is partially independent from aging and dementia,^4^, ^5^, ^6^ suggesting that other yet unrecognized factors are at play. Conceivably, cardiovascular autonomic dysfunction may be one of these factors.

Despite clinical evidence of greater dysautonomia in GBA‐PD is well‐established,^4^, ^5^, ^9^, ^10^ instrumental characterization of pattern and severity of cardiovascular and sudomotor autonomic dysfunction is still insufficient. Nonetheless, several reasons support the importance of assessing autonomic dysfunction instrumentally rather than using scales/questionnaires. (1) Dysautonomia is under‐recognized in 50% of PD^11^ and frequently asymptomatic: up to 75% of cases with abnormal instrumental autonomic tests do not report any complaint.^12^ On the other hand, over 30% of PD patients report orthostatic intolerance without any abnormalities on instrumental testing.^13^ (2) Some autonomic dysfunction (eg, cardiovagal modulation) have poor or no clinical correlates, despite its prognostic relevance on morbidity and survival.^14^, ^15^ (3) Autonomic symptoms could be misdiagnosed on the basis of subjective reporting, which could be misleading. (4) Both symptomatic and asymptomatic dysautonomia are associated with more rapid disease progression and increased the risk of institutionalization, falls, dementia, and sudden cardiac death in PD.^16^, ^17^, ^18^

To date, only a study provided initial evidence of impaired heart‐rate variability and lower vagal modulation in GBA‐PD compared to PD noncarriers, but instrumental assessment was limited to rest and active standing, and sudomotor dysfunction was not explored.^9^ Conversely, the use of standardized operative protocols to extensively assess autonomic dysfunction should be addressed to promote reproducibility and comparability of data among centers and enable finer PD non‐motor phenotyping and prognostic predictions.

In this scenario, this study aimed to provide an extensive clinical and instrumental characterization of both cardiovascular and sudomotor autonomic dysfunction through standardized broadly available autonomic testing protocols in a cohort of well‐characterized GBA‐PD versus matched PD noncarriers. In addition, we explored whether greater dysautonomia proceeds in parallel with more severe cognitive impairment in GBA‐PD. We expected (1) to confirm GBA‐PD patients have more frequent and severe autonomic dysfunction than PD noncarriers^4^, ^5^, ^9^, ^10^ and (2) to find that instrumental assessment of autonomic dysfunction is more sensitive than clinical questionnaires, implying that clinical assessment alone may underestimate an underlying cardiovascular autonomic dysfunction, which could contribute to the reduced survival related to GBA mutations.^4^ We finally expected (3) to find an association between dysautonomia and cognitive dysfunction (reduced frontal‐lobe and/or visuo‐spatial abilities) among carriers, supporting the concept that GBA‐PD present more widespread α‐syn pathology than noncarriers.^4^, ^19^

## Methods

### Study Population

This cross‐sectional study included patients with clinical diagnosis of PD^20^ and carrying heterozygous GBA pathogenic mutations or variants. Exclusion criteria were: (1) comorbidities confounding autonomic testing results or precluding their safe execution (ie, diabetes mellitus, unstable cardiovascular or respiratory disorders, renal failure, alcoholic abuse, current use of medications targeting the autonomic system (eg, adrenergic α‐ or β‐blockers, anticholinergics, etc.), vagal nerve stimulation, vagotomy); (2) diagnosis of dementia according to MDS criteria^21^, ^22^ or DSM‐V^23^; (3) diagnosis of any other neurological disorder; (4) mutations in other PD‐related genes (*LRRK2*, *parkin*, *PINK1*, *SNCA*); (5) inability to attend at our Institute due to restrictions (eg, COVID‐19 pandemic). Out of 716 patients with parkinsonism screened for GBA mutations from September 1, 2008 to September 30, 2020, we found 80 GBA‐PD individuals. Out of this cohort, 25 GBA‐PD were recruited between July 15 to October 20, 2020. Study flow chart is shown in Fig. S1. Twenty‐five PD without GBA mutations and other exclusion criteria, carefully matched for age, sex and disease duration at assessment, were consecutively recruited during the same period as control group (PD noncarriers). All patients were of Caucasian ethnicity and Italian origin. Due to incomplete instrumental data in three GBA‐PD patients and one noncarrier, only 21 GBA‐PD and 24 PD noncarriers were considered for final analysis.

Details of the genetic analysis are available in the Supplementary methods.^24^

### Clinical Evaluation

All the subjects were evaluated at the Movement Disorder Unit of the Neurologic Institute Carlo Besta (Milan, Italy) by Neurologists experienced in movement disorders. Data were collected on main demographical and clinical information, family history, ongoing drugs and device‐aided therapies for PD. Clinical work‐up, performed for all patients in morning hours and ON‐medication, included assessment of motor and non‐motor symptoms, quality of life and functional impairment through the Italian version of the following internationally‐validated scales: Movement Disorder Society‐Unified Parkinson's Disease Rating Scale (MDS‐UPDRS),^25^, ^26^ Hoehn & Yahr stage (H&Y), Non‐Motor Symptoms Scale (NMSS),^11^, ^27^ REM Sleep Behavior Disorder Screening Questionnaire (RBDSQ),^28^ Composite Autonomic Rating Scale (COMPASS‐31),^29^ 8‐items version of Parkinson's Disease Questionnaire (PDQ‐8),^30^ Activities of daily Living (ADL) and Instrumental ADL (IADL).^31^, ^32^ RBD was not confirmed using polysomnography and it is to be considered “probable RBD” (pRBD). Neuropsychological testing is described in detail in the Supplementary methods.

Patients had clinical, neuropsychological, and autonomic testing on the same day. Patients were instructed to take the usual first Levodopa dose in the morning (8.00 am), then the neurologist performed the clinical scales (9.00 am) followed by the neuropsychological assessment (10.30–12.30 am); finally, patients underwent autonomic testing (1.00–2.00 pm).

### Autonomic Testing

Autonomic function testing was performed for all patients in controlled conditions of temperature and humidity according to standard operating procedures. Before testing, all patients were instructed to stop any coffee, tea or taurine‐containing beverages, and all potential confounding medications for at least five half‐lives, except for dopaminergic therapy (patients were allowed to take the first morning dose of Levodopa and autonomic testing had been performed at least 5 h away from the last levodopa intake).

#### Cardiovascular Autonomic Testing

Heart rate (HR) and beat‐to‐beat systolic blood pressure (sBP) and diastolic blood pressure (dBP) were recorded continuously using the Task Force® Monitor device (CNSystems Medizintechnik AG, Graz, Austria). Thereafter, a standard Ewing's test battery^33^ was used to derive the following parameters: basal cardiovascular parameters during rest; RR Intervals variation and E:I ratio during Deep Breathing (DB); Valsalva Ratio (VR, HR in phase II and IV) and presence of BP overshoot in phase IV during Valsalva Maneuver (VM); variation of sBP, dBP and HR after 3 and 5 min of Hand Grip test (HG); variation from baseline of sBP, dBP and HR during 10 min‐Head‐Up Tilt Test (HUTT) at each minute and 1 min after tilting down. In addition, the following sympathetic indexes (SI) were calculated according to BP variations during VM: SI‐1 (BP Fall during phase 2); SI‐2 (entity of BP Recovery late Phase 2); SI‐3 (BP Recovery late Phase 2 compared with baseline); SI‐4 (Magnitude of phase 4); SI‐5 (Pressure recovery time) and BRSa (SI‐6).^34^ Orthostatic hypotension (OH) was defined as a sustained decrease in sBP > 20 mmHg and/or dBP drop>10 mmHg within 3 min of HUTT^35^, ^36^ or as fall in sBP > 30 mmHg and/or dBP > 15 mmHg in the case of concomitant supine hypertension.^37^ The ∆HR/∆sBP ratio at third minute after tilt‐up was calculated.^38^

Spectral analysis in the frequency domain of HR and BP beat‐to‐beat variability based on current HR variability (HRV) Guidelines were used to derive the high (HF, 0.15–0.40 Hz) and low frequency (LF, 0.04–00.15 Hz) powers of RR variability, as well as their ratio (LF/HF).^39^ Autoregressive method was used to obtain SDNN (Standard Deviation of Normal‐to‐Normal intervals), SDNN Index (reflecting the mean of the standard deviations of all NN intervals for all 5 min segments of the entire recording), SDANN (Standard Deviation of the Average of NN intervals), r‐MSSD (root mean square of successive RR interval difference) and pNN50 (Percentage of the adjacent normal‐to‐normal intervals that differed by ≥50 ms), as spectral short‐term measures of HRV in basal resting conditions for 5 min.^39^

We used the handgrip test (HT) to assess the non‐baroreflex mediated α‐adrenergic response. The HT was performed by asking patients to firmly squeeze a blood pressure monitor applying an isometric force at the 30% of maximal effort for 3 min.

#### Sudomotor Autonomic Testing

Sudomotor function was investigated using the Dynamic Sweat Test (DST), which measures post‐ganglionic sudomotor function by stimulating the skin via iontophoresis at 2 mA for 5′ using pilocarpine.^40^, ^41^ Progressive sweating was recorded for 3 min with high resolution digital camera (Toshiba Camileo X150) and analyzed, through ImageJ software [Rasband, W.S. (1997–2015) ImageJ. National Institutes of Health, Bethesda, MD, USA. http://imagej.nih.gov/ij].^40^, ^42^ We used the following indexes: Sweat (nl) × minutes and Sweat/cm^2^/min.^40^, ^41^

### Statistical Analysis

All the analyses were performed with the software STATA 16 (StataCorp, College Station, TX, USA). The primary aim was to compare PD carriers of GBA mutations to PD noncarriers. Therefore, the sample size was calculated on the primary outcome measure (SDNN index), which is the only index of long‐term time‐domain analysis (related to a deficit in the sympatho‐vagal balance) that significantly differentiated PD patients and healthy control individuals.^43^ Expecting a clinically meaningful minimum mean difference of 10.5 ms in SDNN (standard deviation 14.0), the sample size sufficient to have a power of 80% with a two‐tailed type I error of 10% was estimated to be 42 patients (21 per group). A two‐tailed *P* value < 0.05 indicated statistical significance. The comparison of variables between PD noncarriers and different mutations sub‐groups was addressed as secondary aim. Details about descriptive statistics, regression and correlations analyses are provided in the Supplementary methods.

### Ethics

This study was performed in agreement with the principles of the Declaration of Helsinki and approved by the local Ethics Committee (CE n.85/2021). All patients signed written informed consent.

## Results

### Genetic and Clinical Characteristics

Among GBA‐PD, 14 (n = 14/21; 66.7%) underwent sequencing of exons 8, 9, 10 and 11 of GBA gene, while sequencing of the entire gene was performed sequenced in the remaining 7 patients (33.3%). Eleven patients (52%) carried severe GBA mutations (GBA‐SM; 7 p.L444P, 1 p.F213I, 1 p.W184R, 1 p.T352X, 1 p.D409H), while 10 patients (47.6%) carried mild GBA mutations or risk variants (GBA‐MM; 5 p.N370S, 1 p.N370S + p.E326K, 2 p.E326K, 1 p.R301C, 1 p.T369M).

Demographic, clinical and motor features are reported in Table 1. Patients did not differ for main demographic, clinical and therapeutic features (none was taking any medication to treat OH). No differences emerged according to GBA mutations severity. Motor fluctuations and dyskinesias were more common among GBA‐PD than noncarriers.

**TABLE 1:** Clinic and demographic characteristics of the study population

Features	GBA‐PD (N = 21)	PD Noncarriers (N = 24)	*P*‐value^b^	GBA‐SM (N = 11)	GBA‐MM (N = 10)	*P*‐value^c^	*P*‐value^d^	*P*‐value^e^
Demographical data								
Male gender, N (%)	8 (38.1)	12 (50)	0.55	6 (54.5)	2 (20)	1.00	0.14	0.18
Body mass index (kg/cm^2^)	24.9 ± 2.7	25.2 ± 4.8	0.81	24.5 ± 2.6	25.5 ± 2.7	0.63	0.89	0.41
Positive family history, N (%)	9 (42.9)	1 (4.2)	**0.003**	5 (45.5)	4 (40)	**0.007**	**0.019**	1.00
Education (years)	11.9 ± 2.7	12.3 ± 3.7	0.66	11.4 ± 3	12.5 ± 2.3	0.45	0.90	0.35
Clinical data								
Age at onset (years)	45 ± 6.4	43.9 ± 9.6	0.64	43.4 ± 5.3	46.9 ± 7.3	0.87	0.38	0.22
Onset ≤50 years, N (%)	15 (71.4)	19 (79.2)	0.73	9 (81.8)	6 (60)	1.00	0.39	0.36
Age at assessment (years)	55.9 ± 7.9	54.8 ± 10.9	0.69	54.1 ± 5.9	59.1 ± 8.4	0.63	0.27	0.081
Disease duration (years)	10.9 ± 4.1	10.7 ± 5.0	0.86	9.7 ± 3.5	12.2 ± 4.5	0.58	0.41	0.17
Daily dopaminergic therapy								
Levodopa Dosage (mg/day)	335 ± 207	334 ± 274	0.82^f^	301 ± 188	373 ± 230	0.79^f^	0.055^f^	0.14^f^
LEDD‐DA (mg/day)	141 ± 140	130 ± 136	0.79^f^	107 ± 119	179 ± 157	0.63^f^	0.15^f^	0.15^f^
LEDD Total (mg/day)^a^	833 ± 345	658 ± 501	0.19^f^	784 ± 369	888 ± 326	0.14^f^	0.19^f^	0.58^f^
Advanced stage therapy, N (%)	8 (38.1)	6 (25)	0.52^f^	5 (45.5)	3 (30)	0.21^f^	0.77^f^	0.26^f^
DBS‐STN, N (%)	6 (28.6)	6 (25)	0.95^f^	4 (36.4)	2 (20)	0.46^f^	0.42^f^	0.36^f^
DLI, N (%)	2 (9.5)	0 (0)	0.99^f^	1 (9.1)	1 (10)	0.99^f^	1.00^f^	0.53^f^
Duration (years)	7.7 ± 4.7	9.5 ± 3.1	0.45^f^	5.4 ± 1.7	11.7 ± 6.0	**0.026** ^f^	0.48^f^	0.48^f^
MDS‐UPDRS scale and Hoehn&Yahr stage								
Total score	47.6 ± 21.1	44.4 ± 20.3	0.64	50.2 ± 21.7	44.7 ± 21.2	0.29	0.70	0.19
Part I: nM‐EDL	12.2 ± 6.5	9.6 ± 6.4	0.19	12.4 ± 6.9	11.7 ± 6.3	0.18	0.42	0.56
Part II: M‐EDL	12.1 ± 6	10.6 ± 6.6	0.43	12.7 ± 5.8	11.4 ± 6.2	0.21	0.97	0.23
Part III: Motor score (Med‐ON)	17.1 ± 9.5	21.3 ± 10.1	0.13	18.4 ± 9.9	15.6 ± 8.8	0.54	0.064	0.20
Dopaminergic	13.5 ± 7.6	17.4 ± 8.1	0.10	15 ± 7.8	11.9 ± 7.4	0.13	0.055	0.16
Non‐dopaminergic	2.7 ± 2.4	2.9 ± 3.6	0.74	2.6 ± 2.8	2.8 ± 2.0	0.83	0.52	0.60
Speech, N (%)	6 (28.6)	4 (16.7)	0.27	3 (27.3)	3 (30)	0.99	0.59	0.73
Gait, N (%)	5 (23.8)	4 (16.7)	0.49	3 (27.3)	2 (30)	0.20	0.90	0.18
Freezing, N (%)	0 (0)	0 (0)	0.99	0 (0)	0 (0)	0.99	0.99	0.99
Postural instability, N (%)	3 (14.3)	3 (12.5)	0.73	2 (18.2)	1 (10)	0.17	0.56	0.26
Part IV: motor complications	6.1 ± 3.7	3.1 ± 2.9	**0.004**	6.6 ± 3.5	5.7 ± 3.9	**0.002**	0.099	0.29
Dyskinesia	3.0 ± 1.9	1.3 ± 1.4	**0.004**	3.0 ± 1.6	2.9 ± 2.2	**0.002**	**0.026**	0.61
Fluctuations	2.6 ± 2.0	1.4 ± 1.6	**0.034**	2.9 ± 1.9	2.3 ± 2.2	**0.013**	0.30	0.31
Hoehn&Yahr stage	1.8 ± 0.5	1.9 ± 0.6	0.31	1.9 ± 0.5	1.6 ± 0.5	0.87	0.065	0.18
H&Y stage ≥ 3	1 (4.8)	3 (12.5)	0.42	1 (9.1)	0 (0)	0.63	0.99	0.99

*Note*: Data are reported as mean ± standard deviation (SD) or count (percentage). Significant values (*P* < 0.05) are shown in bold.

Abbreviations: LEDD, levodopa equivalent daily dosage; DA, dopamine agonists; DBS: deep brain stimulation; STN, subtalamic nucleus; DLI, duodenal levodopa infusion; nM‐EDL, non‐motor aspects of experiences of daily living; M‐EDL, motor aspects of experiences of daily living.

^a^ Total LEDD: sum of Levodopa Dosage, LED‐DA, LED‐IMAO, LED‐ICOMT and LED for DLI.

^b^ PD noncarriers versus GBA‐PD.

^c^ GBA‐SM versus PD noncarriers.

^d^ GBA‐MM versus PD noncarriers.

^e^ GBA‐SM versus GBA‐MM.

^f^ *P*‐values are calculated according to linear (continuous variables) or logistic (dichotomous variables) regression models adjusted for disease duration.

### Non‐motor Symptoms

GBA‐PD, mainly GBA‐SM, displayed a greater burden of nonmotor symptoms than PD noncarriers according to the NMSS total score, particularly involving sleep (restless legs syndrome, but not pRBD), and pain in GBA‐SM.

Clinical scales and questionnaires did not reveal major differences on items related to autonomic dysfunction between GBA‐PD and noncarriers (MDS‐UPDRS‐IB on Table 1; NMSS and COMPASS‐31 on Table 2). Nevertheless, despite the limited sample size, GBA‐SM described significantly more nocturia, pain and sweating dysfunction than noncarriers (Table 2).

**TABLE 2:** Non‐motor symptoms and autonomic dysfunction

Variables^c^	GBA‐PD (N = 21)	PD Noncarriers (N = 24)	*P*‐value^d^	GBA‐SM (N = 11)	GBA‐MM (N = 10)	*P*‐value^e^	*P*‐value^f^	*P*‐value^g^
NMSS^c^								
Total score	64.5 ± 37.2	40.7 ± 28.4	**0.023**	68.6 ± 41.3	60 ± 33.8	**0.015**	0.20	0.093
Cardiovascular domain score (OH/Syncope)	1.8 ± 3.1	0.8 ± 1.6	0.17	2.5 ± 3.4	1.0 ± 2.5	**0.040**	0.82	0.15
Pts with OH, N (%)	2 (9.5)	1 (4.1)	0.47	0	2 (20)	0.49	0.14	0.12
Pts with symptomatic OH, N (%)	0	0	‐	‐	0	‐	‐	‐
Sleep/Fatigue domain score	16.0 ± 9.7	11.9 ± 8.3	0.15	16.8 ± 9.7	15.0 ± 10.2	0.11	0.47	**0.035**
EDS/Fatigue	7.2 ± 4.8	8.1 ± 4.6	0.47	7.0 ± 4.8	7.3 ± 5.0	0.65	0.53	0.66
Sleep disturbances	5.0 ± 4.6	2.7 ± 3.4	0.068	4.8 ± 3.9	5.1 ± 5.5	0.11	0.14	0.67
Restless leg syndrome	3.8 ± 5.0	1.1 ± 2.4	**0.029**	4.9 ± 5.0	2.6 ± 5.0	**0.006**	0.35	0.059
Mood domain score	12.9 ± 11.9	7.6 ± 8.7	0.097	15.3 ± 13.0	10.3 ± 10.6	**0.031**	0.59	0.058
Apathy	3.1 ± 4.6	1.3 ± 1.0	0.12	3.7 ± 4.3	2.5 ± 5.0	0.055	0.46	0.16
Anxiety	4.0 ± 3.9	2.3 ± 2.7	0.097	3.7 ± 4.0	4.3 ± 4.0	0.17	0.14	0.82
Depression/Anhedonia^b^	5.8 ± 6.5	4.0 ± 5.6	0.37	7.8 ± 7.6	3.5 ± 4.3	0.082	0.65	**0.032**
Perceptual domain score	2.6 ± 4.3	1.8 ± 3.9	0.54	1.5 ± 3.2	3.6 ± 5.4	0.84	0.43	0.45
Hallucinations	1.1 ± 2.3	0.4 ± 0.9	0.19	1.3 ± 2.5	0.9 ± 2.0	0.13	0.38	0.65
Psychosis	0.7 ± 2.6	0.4 ± 1.7	0.65	0.1 ± 0.3	1.4 ± 3.7	0.71	0.45	0.57
Cognition domain score	5.2 ± 6.2	3.7 ± 4.5	0.36	5.0 ± 5.5	5.4 ± 7.2	0.45	0.52	0.81
Attention	2.4 ± 3.5	2.0 ± 2.8	0.70	2.5 ± 2.9	2.3 ± 4.2	0.66	0.95	0.58
Memory	2.8 ± 3.7	1.6 ± 2.3	0.22	2.4 ± 3.7	3.1 ± 4.2	0.36	0.27	0.91
Gastrointestinal domain score	4.2 ± 3.5	3.0 ± 3.3	0.31	5.1 ± 5.0	3.2 ± 3.4	0.12	0.84	0.21
Sialorrhea	0.6 ± 1.1	0.3 ± 0.9	0.47	0.4 ± 0.9	0.7 ± 1.2	0.66	0.48	0.97
Dysphagia	0.9 ± 1.2	0.7 ± 1.4	0.63	1.0 ± 1.3	0.7 ± 1.25	0.35	0.90	0.36
Constipation	2.8 ± 3.7	2.0 ± 2.4	0.39	3.6 ± 3.7	1.8 ± 3.7	0.12	0.96	0.26
Urinary domain score	6.5 ± 5.7	5.9 ± 8.4	0.83	5.4 ± 5.5	7.7 ± 6.0	0.95	0.80	0.92
Urgency	2.2 ± 3.5	2.3 ± 3.7	0.86	1.2 ± 2.4	3.3 ± 4.3	0.49	0.76	0.45
Frequency	0.5 ± 1.4	1.6 ± 3.2	0.14	0.1 ± 0.3	0.9 ± 1.9	0.15	0.49	0.13
Nocturia	3.8 ± 3.8	2.0 ± 2.9	0.095	4.2 ± 3.8	3.5 ± 3.8	**0.046**	0.36	0.25
Sex domain score	3.4 ± 3.9	2.0 ± 4.0	0.26	3.3 ± 3.1	3.6 ± 4.8	0.34	0.45	0.71
Libido	2.4 ± 3.2	1.3 ± 2.8	0.25	2.0 ± 2.7	2.8 ± 3.8	0.51	0.25	0.74
Sexual dysfunction	1.0 ± 2.7	0.7 ± 2.1	0.70	1.3 ± 2.8	0.8 ± 2.5	0.44	0.81	0.33
Miscellaneous domain score	12.0 ± 7.1	5.8 ± 4.8	**0.002**	13.5 ± 7.9	12.2 ± 6	**0.001**	**0.033**	0.16
Pain	3.4 ± 4.0	1.8 ± 3.2	0.16	4.7 ± 4.0	1.9 ± 3.7	**0.034**	0.81	0.13
Hyposmia/hypogeusia	4.8 ± 4.0	2.6 ± 3.3	0.050	5.2 ± 4.3	4.5 ± 3.9	0.062	0.19	0.45
Weight change	0.7 ± 2.2	0.1 ± 0.2	0.17	0.1 ± 0.3	1.3 ± 3.0	0.54	**0.048**	0.17
Excessive sweating	3.1 ± 3.5	1.3 ± 2.2	0.055	3.5 ± 3.2	2.5 ± 3.8	**0.019**	0.32	0.30
COMPASS‐31^c^								
Total score	25.7 ± 14.6	24.2 ± 16.1	0.76	28.2 ± 13.3	22.9 ± 16.2	0.45	0.86	0.35
Cardiovascular domain score	9.3 ± 11.3	9.6 ± 9.9	0.96	10.5 ± 9.0	8.0 ± 13.7	0.84	0.80	0.67
Vasomotor domain score	0.4 ± 0.8	0.4 ± 1.1	0.94	0.4 ± 0.8	0.4 ± 0.9	0.92	0.76	0.55
Urinary domain score	5.1 ± 3.6	4.0 ± 3.9	0.42	4.7 ± 3.6	5.6 ± 3.8	0.15	0.85	**0.031**
Gastrointestinal domain score	6.9 ± 3.9	7.2 ± 3.9	0.79	8.0 ± 4.0	5.6 ± 3.5	0.57	0.35	0.15
Sudomotor domain score	2.2 ± 2.6	1.6 ± 2.0	0.35	2.6 ± 2.8	1.7 ± 2.4	0.45	0.38	0.61
Pupillomotor domain score	1.8 ± 1.3	1.5 ± 1.4	0.38	2.0 ± 1.1	1.7 ± 1.6	0.25	0.67	0.75
RBDSQ^c^								
Total score	4.8 ± 3.2	4.5 ± 2.8	0.78	5.3 ± 3.6	4.3 ± 2.8	0.47	0.72	0.15
Presence of pRBD, N (%)^a^	5 (23.8)	7 (29.2)	0.61	4 (36.4)	1 (10)	0.61	0.18	0.086
PDQ‐8^c^								
Total score	11.1 ± 5.7	7.5 ± 5.0	**0.027**	12.6 ± 6.2	9.4 ± 4.9	**0.005**	0.47	**0.046**
Mobility	1.6 ± 1.4	1.0 ± 1.5	0.19	1.9 ± 1.5	1.3 ± 1.2	0.063	0.85	0.24
Personal care	1.4 ± 1.3	0.9 ± 1.1	0.15	1.3 ± 1.1	1.5 ± 1.6	0.17	0.31	0.84
Depression	1.3 ± 1.2	1.1 ± 1.1	0.69	1.5 ± 1.4	1.0 ± 1.1	0.29	0.65	0.17
Social support	0.6 ± 1.2	0.3 ± 0.7	0.46	0.6 ± 1.4	0.5 ± 1.0	0.41	0.56	0.62
Cognition	0.8 ± 1.0	1.1 ± 1.0	0.37	1.0 ± 1.0	0.6 ± 1.0	0.83	0.20	0.37
Communication	1.9 ± 1.1	1.0 ± 1.3	**0.026**	2.3 ± 0.9	1.4 ± 1.2	**0.002**	0.64	**0.024**
Bodily discomfort	2.0 ± 1.2	0.7 ± 1.2	**0.001**	2.0 ± 1.3	2.0 ± 1.2	**0.008**	**0.012**	0.60
Stigma	1.6 ± 1.3	1.2 ± 1.2	0.36	2.0 ± 1.3	1.1 ± 1.1	0.10	0.78	**0.044**

*Note*: Significant values (*P* < 0.05) are shown in bold.

Abbreviations: OH, orthostatic hypotension; EDS, excessive daytime sleepiness; pRBD, proboable REM‐behavior disorder (polysomnography not performed); NMSS, non‐motor symptoms scale; COMPASS‐31, composite autonomic symptom score‐31; RBDSQ, REM‐behavior disorder screening questionnaire; PDQ‐8, Parkinson's disease questionnaire‐8 items.

^a^ Presence of RBD was defined for a RBDSQ total score ≥6, as reported in.^24^

^b^ Depression/Anhedonia score was calculated with the sum of depression and anhedonia subitems of NMSS.

^c^ Data are reported as mean ± SD or count (percentage). All *P*‐values are calculated according to linear (continuous variables) or logistic (dichotomous variables) regression models adjusted for disease duration.

^d^ PD noncarriers versus GBA‐PD.

^e^ GBA‐SM versus PD noncarriers.

^f^ GBA‐MM versus PD noncarriers.

^g^ GBA‐SM versus GBA‐MM.

### Quality of Life and Functional Impairment

Quality of life was significantly worse in GBA‐PD than PD noncarriers, mainly due to body discomfort and communication issues. ADL and IADL were similarly impaired, GBA‐SM had greater functional impairment than noncarriers on daily cognitive tasks at PD‐CFRS (Table 2).

### Instrumental Assessment of Autonomic Dysfunction

#### Cardiovascular Reflex Responses

Cardiovascular reflex responses are reported in detail in Table 3 and shown in Fig. S2. At *basal resting conditions* and during *parasympathetic tasks*, we found no major differences in main cardiovascular parameters between GBA‐PD and PD noncarriers. At *HUTT*, GBA‐PD showed greater BP drop at 5 min than noncarriers. OH was observed in two GBA patients. During *VM*, the SI‐2 was relatively more preserved in GBA‐PD than PD noncarriers, while no differences were found for other SI. Responses during isometric handgrip test were similar across groups. Exploring main cardiovagal and sympathetic indexes, VR, RR interval variation during DB and E:I ratio showed a progressive decline from PD noncarriers to GBA‐SM (Fig. S2).

**TABLE 3:** Cardiovascular and sudomotor autonomic testing

Cardiovascular parameters^a^	GBA‐PD (N = 21)	PD noncarriers (N = 24)	*P*‐value^b^	GBA‐SM (N = 11)	GBA‐MM (N = 10)	*P*‐value^c^	*P*‐value^d^	*P*‐value^e^
Basal cardiovascular parameters								
Basal RR interval variation	14.3 ± 6.8	15.7 ± 9.3	0.59	12.7 ± 4.6	16 ± 8.5	0.22	0.76	0.26
Basal LF/HF supine	0.9 ± 0.6	0.9 ± 0.7	0.97	1.0 ± 0.8	0.8 ± 0.3	0.85	0.90	0.72
Cardiac parasympathetic parameters								
E:I ratio	1.3 ± 0.2	1.3 ± 0.2	0.52	1.2 ± 0.2	1.3 ± 0.2	0.21	0.97	0.49
%∆E‐I (DB vs. basal) (%)	68.9 ± 90.8	80 ± 102	0.71	69.3 ± 61.8	68.5 ± 118.7	0.76	0.74	0.96
DB RR interval variation	28.7 ± 18.9	31.4 ± 18.9	0.67	30.1 ± 20.4	27.3 ± 18	0.69	0.70	0.96
Valsalva ratio (VR)	1.4 ± 0.2	1.6 ± 0.4	0.058	1.5 ± 0.3	1.3 ± 0.1	0.44	**0.024**	0.12
Cardiovascular sympathetic parameters								
Valsalva maneuver								
SI 1 (Fall during phase 2)	−18.2 ± 14.5	−11.5 ± 13.1	0.13	−20.2 ± 15.8	−16.0 ± 13.5	0.12	0.40	0.97
SI 2 BP recovery late Phase II	10 ± 13.9	−6.8 ± 15.3	**0.04**	9.7 ± 24.9	4.7 ± 14.3	**0.028**	0.062	0.61
Novak Index (SI3)	−14.0 ± 15.5	−18.9 ± 15.8	0.32	−15.5 ± 13.2	−12.3 ± 18.4	0.56	0.32	0.67
Magnitude of phase 4 (SI4)	9.2 ± 10.5	12.5 ± 11.7	0.42	11.3 ± 11.4	7.0 ± 9.5	0.74	0.34	0.59
Pressure recovery time (SI5)	15.5 ± 12.6	13.6 ± 12.6	0.63	17.9 ± 16.5	12.9 ± 6.2	0.43	0.88	0.41
BRSa (SI6)	−1.1 ± 2.0	−2.2 ± 2.6	0.15	−1.2 ± 1.3	−1.0 ± 2.7	0.26	0.26	0.83
Head‐Up Tilt Test (HUTT)								
∆sBP (1 min), mmHg	4.4 ± 11.2	15.7 ± 17.9	**0.016**	5.0 ± 9.9	3.7 ± 13.0	0.093	0.059	0.81
∆dBP (1 min), mmHg	15.2 ± 7.2	19.0 ± 10.7	0.16	14.5 ± 8.1	16.1 ± 6.3	0.27	0.34	0.86
∆HR (1 min), bpm	11.8 ± 7.3	11.6 ± 7.8	0.90	13.5 ± 7.9	9.8 ± 6.4	0.59	0.69	0.33
∆sBP (3 min), mmHg	3.4 ± 12.4	6.0 ± 8.7	0.39	7.3 ± 10.3	−1.1 ± 13.5	0.72	0.074	0.13
∆dBP (3 min), mmHg	8.4 ± 9.7	8.6 ± 8	0.94	10.8 ± 9.1	5.7 ± 10.1	0.48	0.39	0.17
∆HR (3 min), bpm	13.1 ± 7.1	12.0 ± 8.2	0.62	15.6 ± 8.3	10.3 ± 4.2	0.23	0.55	0.084
∆HR/∆sBP index	−0.5 ± 3.9	1.5 ± 4.8	0.15	−1.6 ± 4.5	0.8 ± 2.8	0.087	0.69	0.16
∆sBP (5 min), mmHg	−3.5 ± 11.2	3.5 ± 8.7	**0.027**	−2.9 ± 10.5	−4.1 ± 12.4	0.085	0.078	0.89
∆dBP (5 min), mmHg	1.3 ± 8.2	5.7 ± 8.4	0.092	1.3 ± 9.7	1.3 ± 6.9	0.16	0.23	0.96
∆HR (5 min), bpm	11.8 ± 7.5	11.4 ± 8.8	0.87	14.0 ± 8.0	9.6 ± 6.6	0.51	0.69	0.26
∆sBP (7 min), mmHg	−0.3 ± 7.7	1.3 ± 10.0	0.56	−0.9 ± 7.1	0.3 ± 8.5	0.60	0.95	0.62
∆dBP (7 min), mmHg	2.5 ± 7.2	3.4 ± 9.1	0.71	0.6 ± 7.6	4.3 ± 6.6	0.41	0.53	0.47
∆HR (7 min), bpm	13.2 ± 10.5	11.1 ± 9.6	0.50	13.4 ± 9.7	12.9 ± 11.7	0.54	0.49	0.95
∆sBP (10 min), mmHg	−3.9 ± 8.4	0.7 ± 10.4	0.13	−2.7 ± 6.9	−5.1 ± 9.9	0.38	0.18	0.44
∆dBP (10 min), mmHg	−0.5 ± 6.1	1.4 ± 11.3	0.53	−0.8 ± 5.7	−0.1 ± 6.7	0.45	0.95	0.65
∆HR (10 min), bpm	12.2 ± 9.4	11.1 ± 9.5	0.72	12.2 ± 11.1	12.1 ± 7.9	0.74	0.77	0.94
∆sBP (1 min post Tilt‐down), mmHg	−0.6 ± 16.3	−8.7 ± 18.2	0.14	−2.1 ± 12.2	1 ± 20.5	0.28	0.23	0.94
∆dBP (1 min post Tilt‐down), mmHg	−10.6 ± 12.1	−17.7 ± 12.1	0.060	−10.8 ± 11.9	−10.4 ± 13	0.15	0.11	0.84
∆HR (1 min post Tilt‐down), bpm	−0.2 ± 8.7	−1.0 ± 5.6	0.73	−2.9 ± 6.5	2.7 ± 10.2	0.41	0.15	0.11
Muscular sympathetic parameters								
Handgrip test								
∆dBP (3 min), mmHg	4.6 ± 10.3	6.4 ± 10.7	0.59	3.2 ± 6.9	6.0 ± 13.1	0.37	0.96	0.62
%∆dBP (3 min vs. basal) (%)	6.6 ± 14	8.9 ± 14.2	0.61	3.4 ± 7.9	9.7 ± 18.2	0.25	0.76	0.34
Heart rate variability								
Frequency‐domain analysis								
Basal LF/HF supine	0.9 ± 0.6	0.9 ± 0.7	0.97	1.0 ± 0.8	0.8 ± 0.3	0.85	0.90	0.72
LF/HF (DB)	3.0 ± 3.3	3.3 ± 2.5	0.77	4.2 ± 4.2	1.6 ± 0.7	0.44	0.070	0.72
∆% (LF/HF‐DB vs. LF/HF basal)	265.9 ± 252	349.9 ± 364.5	0.39	388.6 ± 276.4	131 ± 132.8	0.78	0.093	**0.008**
LF/HF (Tilt 1 min)	1.1 ± 0.8	1.2 ± 1.0	0.41	1.3 ± 1.0	0.9 ± 0.4	0.93	0.26	0.28
LF/HF (Tilt 3 min)	2.5 ± 5	2.4 ± 2.7	0.91	3.9 ± 6.7	1.0 ± 0.4	0.33	0.13	0.15
LF/HF (Tilt 5 min)	1.4 ± 1.2	2.2 ± 1.8	0.065	1.7 ± 1.6	1.1 ± 0.5	0.25	0.078	0.50
LF/HF (Tilt 7 min)	1.5 ± 1.9	2.6 ± 1.9	**0.047**	1.9 ± 2.6	1.0 ± 0.6	0.29	**0.014**	0.58
LF/HF (Tilt 10 min)	3.5 ± 9.6	4.3 ± 7.3	0.79	5.7 ± 13.5	1.4 ± 1.3	0.75	0.22	0.53
LF/HF (1 min post‐Tilt down)	1.2 ± 1.4	1.0 ± 0.9	0.67	1.4 ± 1.8	1.0 ± 0.8	0.53	0.90	0.60
Time‐domain analysis								
SDNN (ms)	72.2 ± 6.6	128.0 ± 23.1	**<0.001**	68.8 ± 7.2	75.9 ± 3.0	**<0.001**	**<0.001**	**0.009**
SDANN (ms)	33.3 ± 7.0	108.0 ± 16.8	**<0.001**	31.2 ± 6.2	35.6 ± 7.3	**<0.001**	**<0.001**	0.15
SDNN index (ms)	22.3 ± 4.0	37.1 ± 5.1	**<0.001**	20.8 ± 3.8	24.0 ± 3.6	**<0.001**	**<0.001**	0.068
r‐MSSD (ms)	10.7 ± 2.7	16.1 ± 2.3	**<0.001**	9.3 ± 2.5	12.2 ± 2.0	**<0.001**	**<0.001**	**0.008**
pNN50 (%)	0.7 ± 0.3	1.4 ± 0.2	**<0.001**	0.6 ± 0.3	0.7 ± 0.3	**<0.001**	**<0.001**	0.53
Sudomotor autonomic parameters								
Dynamic sweat test (DST)								
Sweat/Cm^2^/min								
More affected body side (by sweating)	4.5 [1.7–6.4]	7.6 [4.4–14.9]	**0.048**	3.1 [1.7–5.4]	6.1 [4.1–7.0]	0.059	0.19	0.32
Less affected body side (by sweating)	10.0 [6.6–18.0]	13.1 [7.5–34.7]	0.23	7.9 [4.8–30.1]	15.1 [9.6–17.7]	0.19	0.53	0.45

*Note*: See text and supplementary material for further details.

Abbreviations: BRSa, Baroreflex Sensitivity Index‐adrenergic; D, delta; DB, Deep Breathing; DLB, Dementia with Lewy Bodies; dBP, Dyastolic blood pressure; E:I, Expiration:Inspiration; GBA, glucocerebrosidase; HR, Heart Rate; LF/HF, Low Frequency/High Frequency; MM, mild mutations; PD, Parkinson's Disease; PKS, parkinsonism; pNN50, Percentage of successive RR intervals that differ by more than 50 ms; sBP, Systolic blood pressure; SDNN, Standard deviation of NN intervals; SDANN, Standard deviation of the average NN intervals; SI, Sympathetic Index; SM, severe mutations; r‐MSSD, Root mean square of successive RR interval differences; VR, Valsalva Ratio.

^a^ All parameters are reported as mean ± SD, unless otherwise specified. All *P*‐values are calculated according to linear (continuous variables) or logistic (dichotomous variables) regression models adjusted for disease duration. Significant values (*P* < 0.05) are shown in bold.

^b^ PD noncarriers versus GBA‐PD.

^c^ GBA‐SM versus PD Noncarriers.

^d^ GBA‐MM versus PD Noncarriers.

^e^ GBA‐SM versus GBA‐MM.

#### Time‐Domain and Frequency‐Domain Analysis of Heart Rate Variability

**Figure 1:**
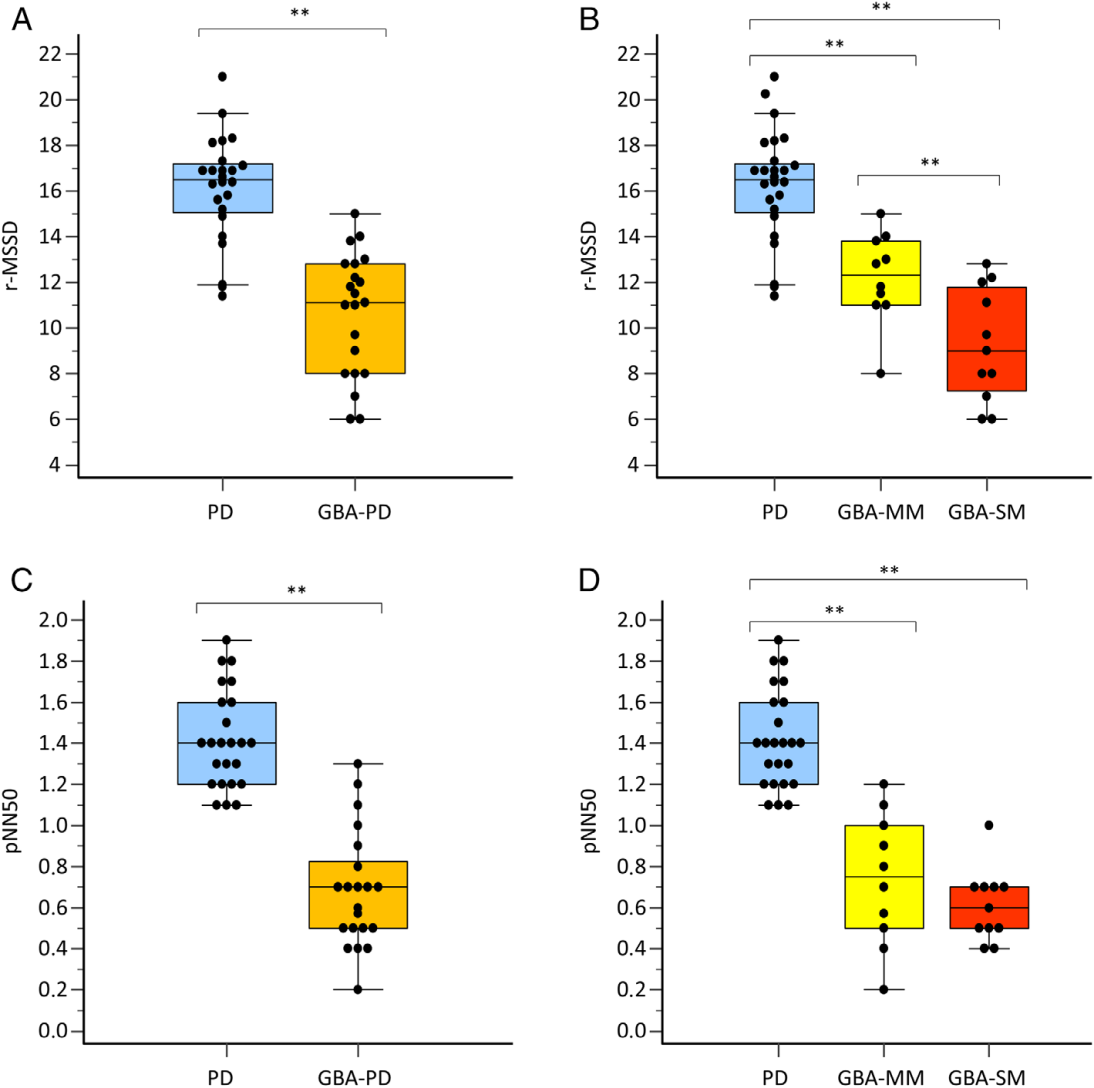
Scatter plots for spectral analyses (r‐MSSD and pNN50) for PD noncarriers (PD) and GBA‐PD (**A**–**C**) and for PD noncarriers, GBA‐SM and GBA‐MM (**B**–**D**), showing 25th and 75th percentile (lower and upper side of the box respectively), the median (middle line) and the minimum to the maximum values (lines extending from box ranges). **P < 0.001.

Spectral analysis of resting HRV demonstrated a high inter‐group discrimination ability, as demonstrated by the coefficient of determination (R2) obtained by regression analysis (Table 3, Fig. 1). SDNN, SDANN, SDNN index, r‐MSSD, pNN50 indexes of HRV were significantly lower in GBA‐PD, GBA‐SM and GBA‐MM compared to PD noncarriers, while SDNN and r‐MSSD significantly differed between GBA‐SM and GBA‐MM.

Concerning frequency‐domain analysis, groups did not differ for LF/HF ratio in basal resting conditions and during DB. During HUTT, the upward trend of LH/HF ratio was less evident for GBA‐PD than noncarriers (Fig. S3). Compared to PD noncarriers, GBA‐PD displayed a lower LF/HF at 7 and 5 min of HUTT, respectively.

#### Sudomotor Autonomic Testing

Dynamic Sweat Testing showed a significantly lower Sweat/Cm^2^/min in GBA‐PD than noncarriers in the most affected body side (Table 3). We found no difference between GBA‐SM and GBA‐MM compared to PD noncarriers and between GBA‐SM vs. GBA‐MM.

### Neuropsychological Assessment

Multiple domain‐MCI was diagnosed in one fourth of GBA‐PD (n = 5/21, 23.8%) while in only 4.2% (n = 1/24) of noncarriers, due to greater impairment in attentive/executive functions (ie, FAB), verbal memory (ie, RAVL recognition task) and visuo‐spatial abilities (Table 4).

**TABLE 4:** Cognitive and neuropsychiatric assessment

Clinical diagnosis	GBA‐PD (N = 21)	PD Noncarriers (N = 24)	*P*‐value^b^	GBA‐SM (N = 11)	GBA‐MM (N = 10)	*P*‐value^c^	*P*‐value^d^	*P*‐value^e^
PD‐MCI, N (%)	7 (33.3)	3 (12.5)	‐	3 (27.3)	4 (40)	‐	‐	‐
PD‐MCI single domain, N (%)	2 (9.5)	2 (8.4)	‐	0 (0)	2 (20)	‐	‐	‐
PD‐MCI multiple domain, N (%)	5 (23.8)	1 (4.2)	‐	3 (27.3)	2 (20)	‐	‐	‐

Cognitive domain		Tests^a^	GBA‐PD (N = 21)	PD Noncarriers (N = 24)	*P*‐value^b^	GBA‐SM (N = 11)	GBA‐MM (N = 10)	*P*‐value^c^	*P*‐value^d^	*P*‐value^e^
Premorbid intelligence		TIB	106.3 ± 8.9	108.9 ± 8.4	0.34	106.1 ± 9.9	106.5 ± 8.4	0.47	0.43	0.90
General cognition		MoCA	23.9 ± 2.4	24.9 ± 2.4	0.33	23.5 ± 2	24.4 ± 4.7	0.11	0.74	0.46
Frontal‐lobe Attentive‐Executive functions		FAB	14.8 ± 2.7	16.5 ± 1.8	**0.020**	14.8 ± 2.8	14.8 ± 2.7	0.061	**0.045**	0.75
		TMT‐A	102.4 ± 194	42.2 ± 21.9	0.17	43.9 ± 28.7	160.9 ± 266.5	0.82	**0.049**	0.15
		TMT‐B	293.5 ± 265	163.2 ± 141.2	0.059	253.8 ± 247.3	333.1 ± 289.1	0.17	0.058	0.64
		TMT‐BA	249.3 ± 267.4	124.6 ± 140.7	0.067	225 ± 254	276.3 ± 294.6	0.13	0.078	0.69
		Digit Span Backward	4.2 ± 0.9	4.7 ± 0.9	0.070	3.8 ± 0.6	4.6 ± 1	**0.011**	0.67	0.11
		Corsi Span Test Backward	3.9 ± 1.2	4.2 ± 1	0.34	4 ± 1	3.7 ± 1.5	0.52	0.31	0.52
		Alternate fluency (Costa)	27.9 ± 16	32.8 ± 13.3	0.28	29.4 ± 15.7	26.4 ± 16.9	0.38	0.35	0.95
		Shifting index (Costa)	0.6 ± 0.3	0.7 ± 0.2	0.20	0.7 ± 0.3	0.6 ± 0.3	0.32	0.24	0.95
Language		Semantic fluency (Costa)	45.5 ± 8.4	46.0 ± 11	0.85	48.1 ± 8.2	42.9 ± 8.2	0.80	0.57	0.24
		Phonemic fluency (Costa)	37.5 ± 13.2	37.6 ± 10.5	0.99	36.9 ± 13.4	38.1 ± 13.8	0.93	0.94	0.68
		Visual nouns denomination test	10 ± 0.2	10 ± 0	0.99	10.0 ± 0.1	9.9 ± 0.3	0.99	0.99	0.99
		Visual verbs denomination test	9.9 ± 0.3	10 ± 0	0.99	9.9 ± 0.3	9.9 ± 0.3	0.99	0.99	0.99
Memory	Verbal memory	Digit Span Forward	5.7 ± 0.8	5.8 ± 1.2	0.81	5.6 ± 0.6	5.7 ± 1	0.72	0.96	0.91
		RAVL immediate recall	44.8 ± 9.9	46.5 ± 7.2	0.55	43.2 ± 10	46.4 ± 10.1	0.36	0.79	0.30
		RAVL delayed recall	10.0 ± 3.2	10.6 ± 2.1	0.51	9.4 ± 3.3	10.6 ± 3.1	0.26	0.97	0.29
		RAVL recognition task	14.5 ± 0.6	14.9 ± 0.3	**0.018**	14.6 ± 0.5	14.4 ± 0.7	0.081	**0.020**	0.34
	Visual memory	Corsi Span Test forward	4.7 ± 1	5 ± 0.9	0.27	4.8 ± 0.9	4.6 ± 1.3	0.43	0.31	0.68
		Benson figure delayed recall	10.8 ± 2.4	10.8 ± 1.5	0.95	10.2 ± 2.1	11.3 ± 2.7	0.38	0.44	0.20
Visuospatial abilities		Clock test	8.5 ± 3.0	9.4 ± 2.2	0.28	8.2 ± 3.0	8.8 ± 3.2	0.22	0.66	0.65
		Benson figure copy	14.9 ± 2.3	16.0 ± 1.1	0.054	15.2 ± 1.1	14.6 ± 3.2	**0.029**	0.12	0.99
**Neuropsychiatric disturbances**		NPI	15.7 ± 13.7	12 ± 11.9	0.37	15.2 ± 12.8	16.3 ± 15.5	0.39	0.50	0.99
		BDI‐II	7.9 ± 4.7	7.3 ± 5.7	0.73	8.5 ± 3	7.2 ± 6.1	0.53	0.96	0.39
		AES	31.6 ± 7.3	27.9 ± 7.1	0.13	34.4 ± 7.9	28.8 ± 5.7	**0.047**	0.81	0.064
Functional Scales		PD‐CFRS	5.2 ± 7.1	2.2 ± 2.5	0.056	5.6 ± 8.3	4.8 ± 6.1	**0.043**	0.12	0.77
		ADL	5.8 ± 0.6	5.8 ± 0.6	0.85	5.8 ± 0.6	5.8 ± 0.6	0.91	0.66	0.81
		IADL, preserved abilities (%)	88.9 ± 25	93.5 ± 19.1	0.56	89 ± 23.3	88.8 ± 27.9	0.40	0.83	0.84

Abbreviations: ADL, activities of daily living; AES, apathy evaluation scale; BDI‐II, back depression inventory‐II; FAB, frontal assessment battery; IADL, instrumental activities of daily living; MoCa, montral cognitive assessment; MCI, mild‐cognitive impairment; NPI, neuropsychiatric inventory; PD‐CFRS, PD‐cognitive functional rating scale; RAVL, ray auditory verbal learning; TIB, Test di Intelligenza Breve (“Brief Intelligence Test”); TMT‐A, B, BA, Trail making test‐A, B, BA.

^a^ All scores are corrected for age and education level and reported as mean ± SD. All *P*‐values are calculated according to linear (continuous variables) regression models adjusted for disease duration. Significant values (*P* < 0.05) are shown in bold. *P*‐values under 0.1 are considered as trends for significance and are shown in italics.

^b^ PD noncarriers versus GBA‐PD.

^c^ GBA‐SM versus PD noncarriers.

^d^ GBA‐MM versus PD noncarriers.

^e^ GBA‐SM versus GBA‐MM.

### Relationship Between Autonomic Dysfunction and Cognitive Impairment

In GBA‐PD, lower E:I ratio was associated with poorer performances in FAB (*r* = 0.57, *P* = 0.009; coefficient of determination *R* ^2^ = 0.32) and Benson Figure Copy (*ρ* = 0.51, *P* = 0.021; *R* ^2^ = 0.18) and greater functional impairment at PD‐CFRS (*ρ* = −0.58, *P* = 0.006; *R* ^2^ = 0.22), while VR correlated with Benson figure copy (*ρ* = 0.49; *P* = 0.039; *R* ^2^ = 0.14) and RAVL recognition task (*ρ* = 0.53; *P* = 0.022; *R* ^2^ = 0.21). In PD noncarriers, E:I ratio positively correlated with Benson figure Copy scores (*r* = 0.44, *P* = 0.030; *R* ^2^ = 0.20), but no associations emerged between E:I ratio and FAB and between VR and all cognitive tests. Compensatory rise of HR at 3 min of HUTT was negatively associated with FAB scores in GBA‐PD (*ρ* = −0.55; *P* = 0.015; *R* ^2^ = 0.16) and not in PD noncarriers. Similar results were obtained after stratifying both groups according to median scores of cognitive tests and to diagnosis of PD‐MCI (Supplementary Results).

Cognitive test performance was not associated with spectral indexes, sympathetic indexes, BP responses during HUTT and isometric test in both groups.

## Discussion

We extensively investigated cardiovascular autonomic function and, for the first time, sudomotor function in a cohort of GBA‐PD and matched PD control subjects. In particular, we provided the instrumental correlate of greater cardiovascular and sudomotor dysautonomia in GBA‐PD and outlined a specific pattern of sympathetic and cardiovagal dysregulation. Moreover, we demonstrated a clear mismatch between subjective reporting of symptoms and objective instrumental findings, supporting the appropriateness of implementing autonomic testing in clinical practice.

### Profile of Cardiovascular Autonomic Dysfunction

#### Cardiovagal Function

Parasympathetic function during active maneuvers was similarly affected in GBA‐PD and PD noncarriers. Nevertheless, spectral measures of HRV were severely impaired in GBA‐PD compared to PD noncarriers. Some spectral indexes (r‐MSSD and SDNN) proved so robust to even differentiate GBA‐PD according to mutation type (PD noncarriers < GBA‐MM < GBA‐SM). Normal HRV in long‐term (at least 24‐h) recordings is an index of good cardiovascular health and overall autonomic integrity.^44^, ^45^ Nevertheless, short‐term measurements are more standardized due to less susceptibility to environmental influences, so being able to more selectively reflect cardiac sympatho‐vagal modulation.^46^, ^47^ In line with recent evidences,^9^ our results confirm a dysfunctional sympatho‐vagal cardiac modulation during rest in GBA‐PD, appearing before the efficiency of active compensatory responses is lost. The relatively younger age of our GBA‐PD cohort further strengthens the relevance of our data and the likelihood of replication in independent cohorts, as an age‐related decline of HRV measures is physiologically expected.^44^ Importantly, HRV carries a remarkable prognostic and predictive role: low HRV is independently associated with increased risk of sudden cardiac death^18^ and overall mortality^14^, ^48^ and it is associated with an increased risk of PD in the general population.^49^ Furthermore, reduction of HRV is a relative early finding (being reported in idiopathic RBD^50^ and at different PD stages^45^, ^47^, ^51^, ^52^) that predicts the development and severity of OH.^51^, ^52^

#### Sympathetic Function

Our data suggest a similar cardiac sympathetic function in GBA‐PD and PD noncarriers during active maneuvers, along with a relative preservation of baroreflex‐mediated vasoconstrictive α‐adrenergic responses (SI‐2 index) in GBA‐PD.^34^ Despite this, compared to PD noncarriers, GBA‐PD showed a clearly distinct pattern of baroreflex modulation during HUTT, characterized by a milder increase of LF/HF ratio. An increase in LF/HF ratio is normally expected upon orthostatic stress^53^ indicating a prompt sympatho‐vagal responsiveness to standing. In patients with unexplained syncope, decrease in LF/HF ratio in the first 5 min of HUTT predicts syncope versus normal test.^54^ Accordingly, GBA‐PD displayed larger orthostatic sBP drops compared to PD noncarriers. Evidences show that BP compensatory responses in early HUTT rely on sympathetic‐induced vasoconstriction,^34^ while BP values maintenance over time requires a combination of cardiovascular sympathetic responses and cardiovagal modulation.^30^ In line with this, our results suggest that a more severe cardiac sympatho‐vagal demodulation in GBA‐PD, along with relative sparing of peripheral vasoconstrictive responses, is responsible for inadequate increase of LH/HF and poor BP maintenance during HUTT.^55^ Overall, considering that sympathetic cardiac impairment and reduction in HRV are hallmarks of efferent baroreflex failure in synucleinopathies,^56^ our results demonstrate a greater efferent baroreflex insufficiency in GBA‐PD compared to PD noncarriers. Some studies suggested a potential effect of Levodopa in lowering BP during HUTT and interfering on sympathetic‐parasympathetic regulation during orthostasis, but conclusive results are lacking.^57^ Despite a confounding effect of dopaminergic therapy is not completely excludible, our study groups were all uniform for total LEDD and autonomic testing was performed for all patients at least 5 h after the last levodopa intake. Moreover, the ON‐medication state is more suitable for common clinical practice, as many patients are not able to tolerate a prolonged study protocol in the medication‐OFF state.

### Sudomotor Autonomic Dysfunction

Post‐ganglionic sweat output was significantly more impaired in GBA‐PD than PD noncarriers, with a seemingly selective involvement of the most affected body side. In PD, sweating dysfunction displays a length‐dependent progression, resulting in hypo‐anhidrotic areas at distal sites and compensatory axial sweating.^58^, ^59^ At skin biopsy, PD displays intraepidermal nerve fiber denervation and loss of autonomic nerves to blood vessels and sweat glands, consistent with somatic and autonomic small fiber neuropathy.^43^, ^60^ α‐syn deposits in autonomic fibers, including around sweat glands, are more abundant in PD with autonomic failure.^41^, ^43^ Reduced sweat production in GBA‐PD could suggest a greater degeneration of autonomic fibers directed to sweat glands on more affected body side. Accordingly, α‐syn deposits in autonomic fibers have been detected from skin biopsy in GBA‐PD,^61^, ^62^ despite the role of skin α‐syn deposits is still unclear^63^ and further studies are needed.

### Is Cardiovascular Dysautonomia Associated with Concomitant Cognitive Dysfunction?

We found a positive association between worse cognitive performances and parasympathetic dysfunction, particularly the E:I ratio. However, this was not specific to GBA‐PD, because the E:I ratio was positively associated to visuo‐spatial abilities in both carriers and noncarriers.

Nevertheless, only GBA‐PD displayed a significant association between the E:I ratio and FAB scores as well as concomitant MCI. This is consistent with previous studies showing an association between reduced parasympathetic measures (E:I ratio, 30:15 ratio and r‐MSSD index) and greater risk for MCI in PD.^64^, ^65^ Notably, decreased parasympathetic activity is associated with worse cognitive performance both in general population without dementia^66^ and in Alzheimer's disease,^67^ suggesting a synucleinopathy‐independent association between parasympathetic function and cognition. Greater autonomic dysfunction in GBA‐PD could actively exacerbate cognitive deficits, with main regards to executive functions. Common underlying pathophysiology should be considered,^68^ in terms of hemodynamic‐related effects (eg, impaired cerebral autoregulation causing white matter lesions),^68^, ^69^ or neuropathological links (eg, brainstem autonomic nuclei degeneration causing noradrenergic and cholinergic denervation of prefrontal and anterior cingulate cortices),^68^, ^70^ or both.

### Limitations and Strengths

We acknowledge that our study has limitations, the most relevant being the small sample size.

Therefore, further study in larger cohorts of GBA‐PD versus PD noncarriers, additionally including patients with dementia with Lewy bodies and non‐PD healthy control groups, is needed to replicate the present findings. Moreover, autonomic testing had been performed without stopping DA agonists and Levodopa for at least five half‐lives as we did for other concomitant medications potentially impacting autonomic testing but limited to 24 h after the last DA agonists intake and at least 5 h away from the last levodopa intake. Levodopa was not stopped because its pharmacodynamic effects (long‐duration response) last much longer than five half‐lives.^71^ Some nonmotor and autonomic symptoms were not objectively assessed, and we did not provide data on MIBG myocardial scintigraphy and skin biopsy. Finally, we acknowledge that the DST has been used on a limited cohort of PD patients so far as compared to other tests used to investigate the sudomotor function, such as the “QSART” and the “TST”. The DST has been recently applied to differentiate PD from MSA,^41^ and it may be more widely used in clinical practice because its equipment is the least expensive.

Despite such limitations, there are strengths worth emphasizing. First, this is the first study providing a comprehensive instrumental assessment of both cardiovascular and sudomotor autonomic function in a well‐characterized cohort of GBA‐PD, studied with standardized operative protocols and in reproducible conditions and compared to carefully matched PD controls. In confirmation, despite small sample size, highly significant differences (*P* < 0.001) were obtained for all time‐domain spectral data analyses, even when limiting analysis to severe versus mild *GBA* mutations, emphasizing the strong effect of *GBA* mutations on cardiovascular autonomic function, particularly on HRV.

## Conclusions

The present study demonstrates that GBA‐PD display more severe cardiovascular autonomic impairment, mainly due to greater cardiac sympathetic and parasympathetic demodulation and impaired efferent baroreflex, along with a more pronounced sudomotor dysfunction. Critically, clinical correlates of these autonomic abnormalities could be minimal or absent, despite their potential impact on morbidity (eg, falls and fractures) and mortality. Translated into clinical practice, we suggest assessing cardiovascular autonomic reflexes instrumentally in all GBA‐PD patients (eg, cardiovascular autonomic tests, prolonged ambulatory test for delayed OH, 24 h ECG monitoring and/or short‐recordings of HRV whenever available), even in the absence of clinical complaints and particularly in younger individuals, whose compensatory mechanisms could mask or delay any symptom. We also encourage to use standardized autonomic protocols to promote comparability of data among centers. Finally, in a “*precision medicine*” perspective, cardiovascular autonomic measures (mainly HRV) should be included as outcome measures in disease‐modifying clinical trials and in future prospective studies investigating survival in GBA‐PD.

## Author Roles

(1) Research Project: A. Conception, B. Organization, C. Execution; (2) Statistical Analysis: A. Design, B. Execution, C. Review and Critique; (3) Manuscript Preparation: A. Writing of the First Draft, B. Review and Critique.

G.D.: 1A, 1C, 2A, 2C, 3A, 3B

G.S.: 1A, 1C, 2A, 2C, 3A, 3B

E.C.: 1A, 2A, 2B, 3B

B.G.: 1C, 3A

A.F.: 1C

A.E.E.: 3B

S.H.M.J.P.: 1B, 3B

S.P.: 1B, 3B

P.A.: 3B

F.I.: 1C

N.G.A.: 3B

L.M.R.: 3B

R.E.: 1B, 3B

R.C.: 1A, 1B, 1C, 2A, 2C, 3A, 3B

## Disclosures

**Ethical Compliance Statement:** We confirm that we have read the Journal's position on issues involved in ethical publication and affirm that this work is consistent with those guidelines. Ethics Committee of the Fondazione IRCCS Istituto Neurologico Carlo Besta, Milano (reference number: CE n.85/2021). The study was conducted in accordance with the declaration of Helsinki and written informed consent was obtained by study participants.

**Funding Sources and Conflicts of Interest:** All the authors report no conflict of interest related to this manuscript.

**Financial Disclosures for Previous 12 Months:** RC has received speaking honoraria from Zambon Italia; Zambon SAU; Bial Italia Srl; Advisory board fees from Bial; Research support from the Italian Ministry of Health; Editor‐in‐Chief of the neuromuscular and movement disorders section of Brain Sciences; Associate Editor of Parkinsonism and Related Disorders, Frontiers in Neurology and Frontiers in Ageing Neuroscience. EC has received speaking honoraria from Zambon Italia. All other authors report no financial disclosures.

## Supporting information


**Data S1.** Supplementary material.

**Data S2.** Supplementary material.

**Figure S1.** Flow‐chart of the study.

**Figure S2.** Scatter plots for E:I ratio, RRI variation during DB and VR ratio values for PD noncarriers (PD) and GBA‐PD (**A**, **C**, **E**) and for PD noncarriers, GBA‐SM, and GBA‐MM (**B**, **D**, **F**), showing 25th and 75th percentile (*lower* and *upper* side of the box, respectively), the median (middle line) and the minimum to the maximum values (lines extending from box ranges).

**Figure S3.** Scatter plots of LF/HF ratio at 1, 3, 5 and 7 min at HUTT for PD noncarriers (PD) and GBA‐carriers (**A**) and for PD, GBA‐SM, and GBA‐MM (**B**), showing 25th and 75th percentile (*lower* and *upper* side of the boxes, respectively), the median (middle line) and the minimum to the maximum values (lines extending from box ranges), excluding outliers.

**TABLE S1.** Instrumental cardiovascular and sudomotor autonomic assessment in GBA‐L444P (GBA‐SM) and GBA‐N370S (GBA‐MM).

## Data Availability

The datasets used and analyzed during the current study are available from the corresponding author upon reasonable request and after its approval by the Ethics Committee.
