# Supplementary material for: Unraveling Autonomic Dysfunction in GBA‐Related Parkinson's Disease
Source: Mov Disord Clin Pract. 2023 Oct 13;10(11):1620–38. doi: 10.1002/mdc3.13892 (PMC10654845; doi:10.1002/mdc3.13892)
Supplement: Supplementary file 1 — Data S1. Supplementary material. [file MDC3-10-1620-s002.docx]

**Supplementary Material**

**Supplementary Methods**

***Genetic analysis***

Genetic analysis was performed on DNA extracted from peripheral leukocytes. Genetic analysis of GBA gene was performed either sequencing exons 8, 9, 10 and 11 for the most common mutations and variants associated with PD by a multiplex PCR assay or sequencing of the entire gene through Next Generation Sequencing (NGS).[1] All variants were confirmed by conventional Sanger sequencing. Mutations in other PD-related genes and less common GBA variants in PD noncarriers were excluded using NGS in all subjects.

NGS for PD-related genes was performed with Nextera Enrichment Sample Illumina (Illumina), according to the manufacturers’ instructions.

In this case, apart from *GBA,* genes analyzed included *ATP13A2, CHCHD2, DNAJC13, DNAJC6, EIF4G1, FBXO7, GCH1, GRN, GYGYF2, HTRA2, LRP10, LRRK2, MAPT, PARK2, PARK7, PINK1, PLA2G6, POLG, RAB39B, SNCA, SYNJ1, TMEM230, UCHL1, VPS13C, OPA1* and *PTRHD1.* DNA processing and DNA-seq analysis were performed using Illumina MiSeq Sequencer. Annovar (table_annovar.pl) software was used for variants annotation. Mutations were classified as pathogenic if they rarely occurred in healthy individuals (i.e., dbSNP, and 1000 Genomes databases), were predicted to cause alterations to protein sequence with *in silico* prediction software (SIFT, PolyPhen, MutationTaster), or in the case of deleterious effects on protein normal function. All identified variants and genetic regions with coverage less than 30x were further confirmed using Sanger sequencing (primer sequences and PCR conditions are available upon request).

Whereas either method was used to detect GBA-PD, all PD noncarriers underwent sequencing of the entire gene to exclude less common GBA pathogenic variants. Mutations in other PD-related genes were excluded using NGS in all subjects.

***Autonomic testing***

**Valsalva Maneuver**: Cardiovascular Sympathetic parameters during Valsalva maneuver are calculated according to Novak P[2]. In particular SI1 (Fall during phase 2) is calculated as percentage variation between baseline mean BP and minimal pressure in phase II; SI 2 BP Recovery late Phase II is calculated as percentage variation between BP at phase IIb and minimal BP in phase IIa; Novak Index (SI3) derives from percentage difference between mean BP at baseline and at phase IIb; Magnitude of phase 4 (SI4) is calculated as variation between maximum value of mean BP during overshoot and baseline mean BP; Pressure recovery time (SI5 or PRT) corresponds to Time interval between BP drop in phase III and return to BP baseline levels and BRSa (SI6) is calculated as (SI1 + 0.7* SI2)/ PRT

**Head-Up Tilt Test** ∆ of sBP, dBP and HR at each time point of HUTT are calculated with reference to basal sBP, dBP and HR, respectively

**∆HR/∆sBP index (Kauffman Index)** is calculated as ∆HR/∆sBP at third minute of Head-Up Tilt Test compared to basal HR and sBP values (bpm/mmHg).[3]

**Time-Domain Analysis** Heart rate variability time-domain indices are calculated during a monitoring period of 5 minutes in basal resting conditions.[4]

***Neuropsychological assessment***

Neuropsychological assessment was performed by neuropsychologists experienced in movement disorders, blinded to patient genetic status. Internationally validated scales were used to estimate premorbid intelligence level using Brief Intelligence Test (TiB),[5] global cognitive function using the Montreal Cognitive Assessment Scale (MoCA),[6] individual cognitive domains function as follows: *(1) Attention and executive functions*: Frontal Assessment Battery (FAB), Trail Making Test-A (TMT-A), TMT-B, TMT-BA, Digit Span Backward, Corsi Span Test Backward, Costa’s alternate fluency and shifting index;[7-10] *(2) Language*: Costa’s semantic and phonemic fluency, Visual nouns denomination and Visual verbs denomination tasks of E.N.P.A battery (Neuropsychological examination of Aphasia-Italian version);[10,11] *(3) Memory*: Digit Span Forward, Rey Auditory Verbal Learning (RAVL) immediate recall, RAVL delayed recall and RAVL recognition task for verbal memory; Visual Span Forward, Benson figure delayed recall and Benson figure recognition task for visual memory;[9,12,13] *(4) Visuo-spatial abilities*: Benson figure copy and Clock design test;[13,14] *(5)* neuropsychiatric symptoms[15-18] and functional impact of cognitive impairment on daily activities.[19] Scores of neuropsychological tests were expressed as raw, corrected (according to age and education level) and equivalent scores and adjusted for disease duration. PD-Mild Cognitive Impairment (PD-MCI) was diagnosed and classified in single-domain MCI or multiple-domain MCI according to level II of diagnostic criteria for PD-MCI.[20,21]

The Italian version of PD-Cognitive Functional Rating Scale (PD-CFRS) was used to assess the functional impact of cognitive impairment on common daily activities (<https://www.movementscales.com/formulario-func-italian>).

***Statistical analysis***

Descriptive statistics of continuous variables were reported as mean and standard deviation (SD) or median and inter-quartile range (25th–75th percentile [interquartile range, IQR]) according to the normality of distribution, while categorical variables were presented as counts and percentages.

All comparisons of clinical features were performed using regression analysis adjusted for disease duration with linear (continuous with normal distribution) or log (continuous with non-normal distribution [negative binomial distribution]) or logit (dichotomous) link. Specifically, normality of distribution was initially assessed using the Shapiro-Wilk test. Given the exploratory nature of the study and the approach used to address between-group comparisons (primary aim: comparison between PD carriers vs. PD noncarriers; secondary aims: comparisons between PD noncarriers vs. carriers of mild mutations; comparisons between PD noncarriers vs. carriers of severe mutations; comparison between carriers of mild vs. severe mutations), no adjustment for multiple comparisons was considered.

To investigate whether patients with more severe autonomic dysfunction had concomitant cognitive dysfunction, we performed two sets of analyses. First, we investigated the direct correlation (calculating Pearson’s or Spearman’s rank correlation coefficients [r and ρ, respectively] depending on normality of distribution) between main cardiovascular autonomic indexes and cognitive tests (MoCA, FAB, Benson figure copy test, RAVL recognition task and PD-CFRS scores). The coefficient of determination (R^2^) was obtained by regression analysis to explain the entity of the associations found. Then, we analyzed between-group differences in autonomic parameters after stratifying patients (GBA-PD and PD noncarriers, as distinct groups) according to (1) median scores, and (2) normal/abnormal scores of MoCA, FAB and Benson figure copy, and (as well as 3) by the presence vs. absence of PD-MCI.

**Supplementary Results**

**Relationship between autonomic dysfunction and cognitive impairment.**

GBA-PD (but not PD noncarriers) with lower FAB scores displayed significantly lower E:I ratio than GBA-PD with higher FAB scores (p=0.030). E:I ratio was lower in GBA-PD with MCI than cognitively normal GBA-PD (p=0.035), while no differences were found in noncarriers. No differences emerged according to Benson figure copy and MoCA scores in both groups. In all these cases, groups resulting from stratification according to cognitive scales did not differ from age and disease duration at assessment (data not shown).

**References**

1. Straniero L, Rimoldi V, Melistaccio G, Di Fonzo A, Pezzoli G, Duga S, Asselta R. A rapid and low-cost test for screening the most common Parkinson's disease-related GBA variants. Parkinsonism Relat Disord 2020;80:138-141
2. Novak P. Assessment of sympathetic index from the Valsalva maneuver. Neurology 2011;76:2010-6.
3. Norcliffe-Kaufmann L, Kaufmann H, Palma JA, et al. Orthostatic heart rate changes in patients with autonomic failure caused by neurodegenerative synucleinopathies. Ann Neurol 2018;83:522-531.
4. Heart rate variability: standards of measurement, physiological interpretation and clinical use. Task Force of the European Society of Cardiology and the North American Society of Pacing and Electrophysiology. Circulation 1996;93:1043-65.
5. Colombo L, Sartori G, Brivio C. Stima del quoziente intellettivo tramite l’applicazione del TIB (Test Breve di Intelligenza). G Ital Psicol 2002; 613–638.
6. Santangelo G, Siciliano M, Pedone R, et al. Normative data for the Montreal Cognitive Assessment in an Italian population sample. Neurol Sci 2015;36:585-91.
7. Appollonio I, Leone M, Isella V, et al. The Frontal Assessment Battery (FAB): normative values in an Italian population sample. Neurol Sci 2005;26:108-16.
8. Giovagnoli AR, Del Pesce M, Mascheroni S, Simoncelli M, Laiacona M, Capitani E. Trail making test: normative values from 287 normal adult controls. Ital J Neurol Sci 1996;17:305-9.
9. Monaco M, Costa A, Caltagirone C, Carlesimo GA. Forward and backward span for verbal and visuo-spatial data: standardization and normative data from an Italian adult population. Neurol Sci 2013;34:749-54. Erratum in: Neurol Sci 2015;36:345-7.
10. Costa A, Bagoj E, Monaco M, et al. Standardization and normative data obtained in the Italian population for a new verbal fluency instrument, the phonemic/semantic alternate fluency test. Neurol Sci 2014;35:365-72.
11. Capasso R, Miceli G. Esame Neuropsicologico per l'Afasia - E.N.P.A. Springer-Verlag Italia. 2005. www.springer.com/gp/book/9788847001527
12. Carlesimo CA, Caltagirone C, Gainotti G, Nocentini U. Gruppo per la standardizzazione della Batteria per il Deterioramento Mentale. Batteria per la valutazione del deterioramento mentale (parte II): descrizione di uno strumento di diagnosi neuropsicologica. Archivio di psicologia, Neurologia e Psichiatria 1995; 4, 471-488
13. Possin KL, Laluz VR, Alcantar OZ, Miller BL, Kramer JH. Distinct neuroanatomical substrates and cognitive mechanisms of figure copy performance in Alzheimer's disease and behavioral variant frontotemporal dementia. Neuropsychologia 2011;49:43-8.
14. Siciliano M, Santangelo G, D'Iorio A, Basile G, Piscopo F, Grossi D, Trojano L. Rouleau version of the Clock Drawing Test: age- and education-adjusted normative data from a wide Italian sample. Clin Neuropsychol 2016;30:1501-1516.
15. Fernandez HH, Aarsland D, Fénelon G, et al. Scales to assess psychosis in Parkinson's disease: Critique and recommendations. Mov Disord 2008;23:484-500.
16. Schrag A, Barone P, Brown RG, et al. Depression rating scales in Parkinson's disease: critique and recommendations. Mov Disord 2007;22:1077-92.
17. Wang YP, Gorenstein C. Psychometric properties of the Beck Depression Inventory-II: a comprehensive review. Braz J Psychiatry 2013;35:416-31.
18. Leentjens AF, Dujardin K, Marsh L, et al. Apathy and anhedonia rating scales in Parkinson's disease: critique and recommendations. Mov Disord 2008;23:2004-14.
19. Kulisevsky J, Fernández de Bobadilla R, Pagonabarraga J, et al. Measuring functional impact of cognitive impairment: validation of the Parkinson's disease cognitive functional rating scale. Parkinsonism Relat Disord 2013;19:812-7.
20. Litvan I, Goldman JG, Tröster AI, et al. Diagnostic criteria for mild cognitive impairment in Parkinson's disease: Movement Disorder Society Task Force guidelines. Mov Disord 2012;27:349-56.
21. Goldman JG, Holden SK, Litvan I, McKeith I, Stebbins GT, Taylor JP. Evolution of diagnostic criteria and assessments for Parkinson's disease mild cognitive impairment. Mov Disord 2018;33:503-510.
