# Supplementary figures and images for: Unraveling Autonomic Dysfunction in GBA‐Related Parkinson's Disease
Source: Mov Disord Clin Pract. 2023 Oct 13;10(11):1620–38. doi: 10.1002/mdc3.13892 (PMC10654845; doi:10.1002/mdc3.13892)

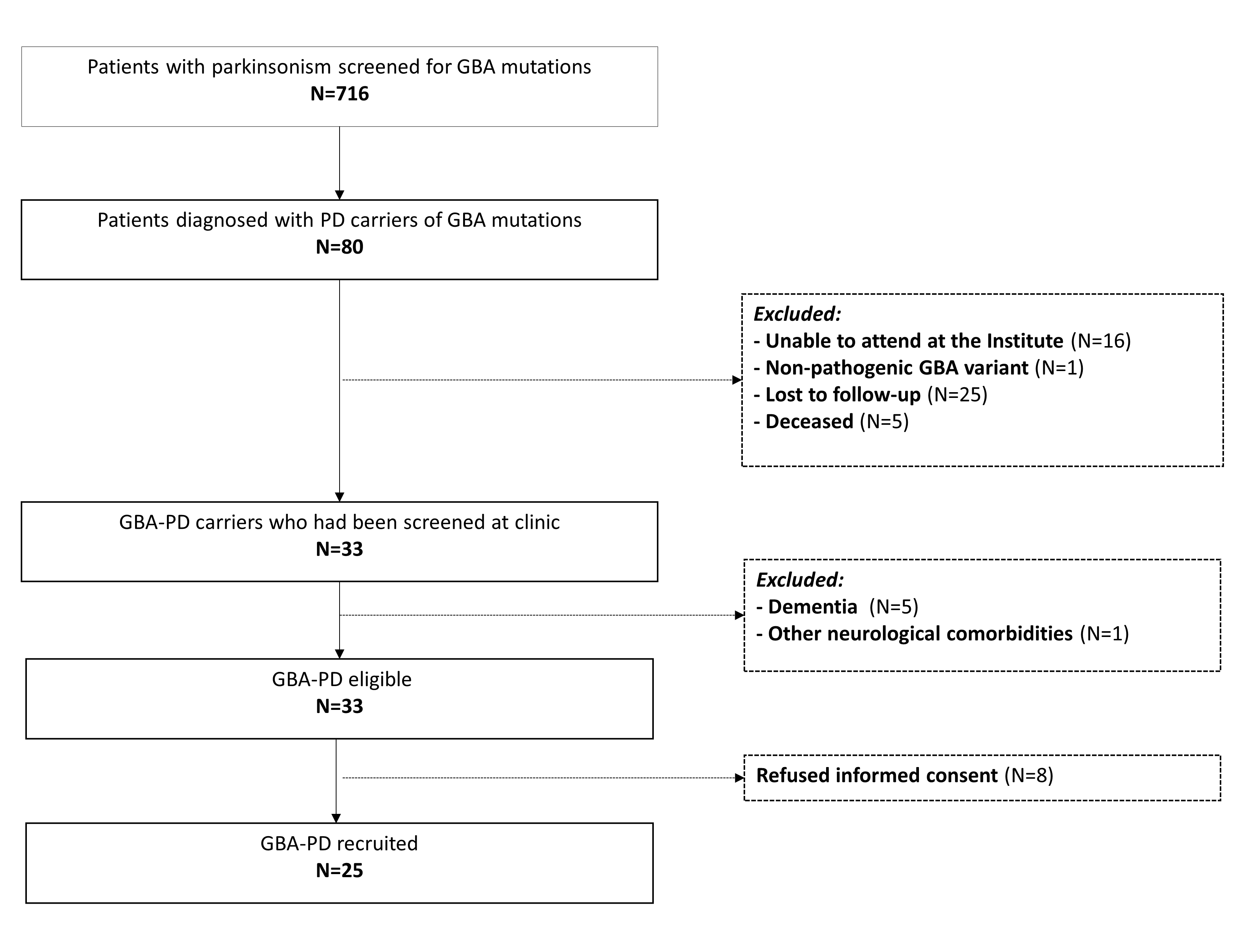

Supplement: Supplementary file 3 — Figure S1. Flow‐chart of the study. [file MDC3-10-1620-s001.tif]

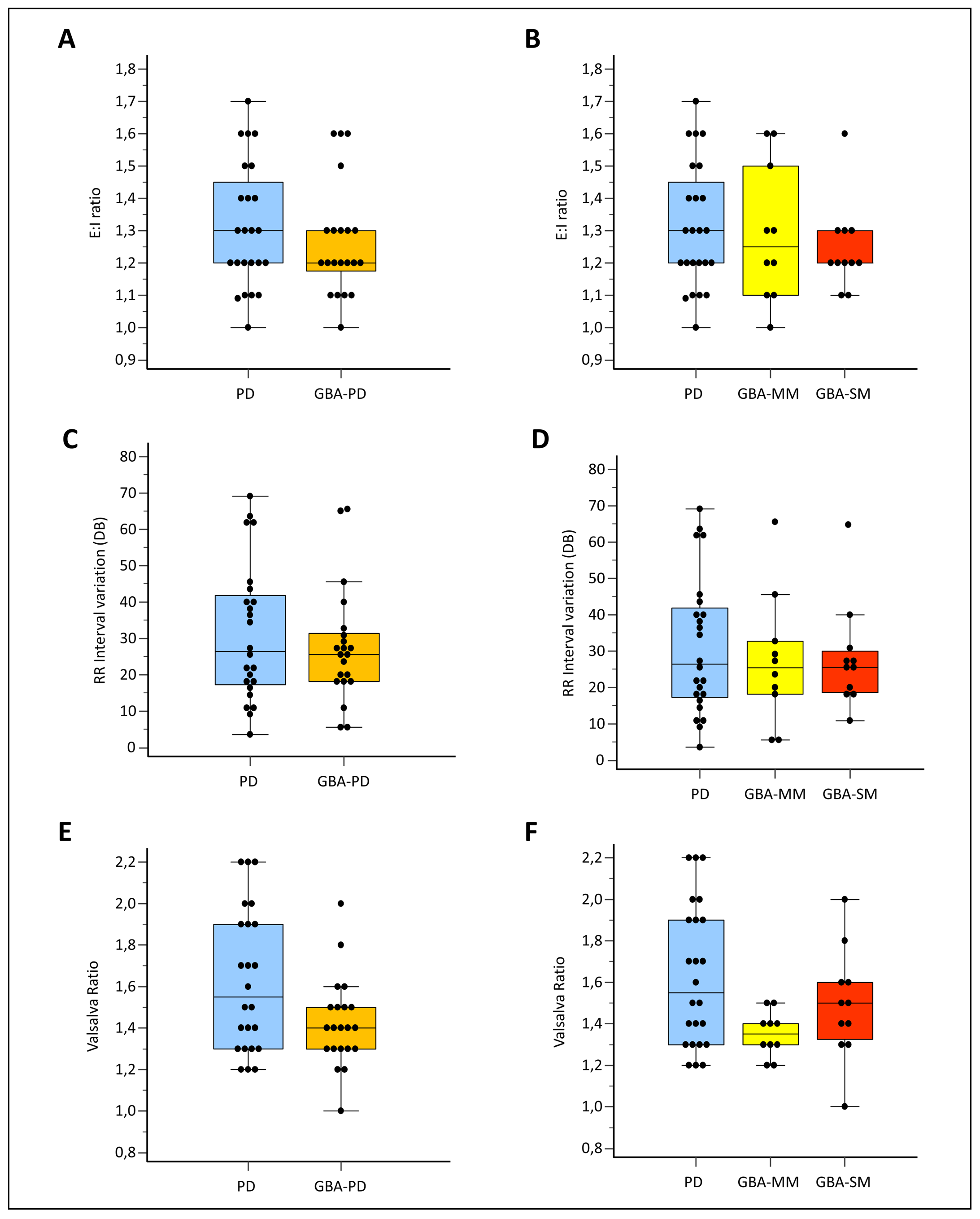

Supplement: Supplementary file 4 — Figure S2. Scatter plots for E:I ratio, RRI variation during DB and VR ratio values for PD noncarriers (PD) and GBA‐PD (A, C, E) and for PD noncarriers, GBA‐SM, and GBA‐MM (B, D, F), showing 25th and 75th percentile (lower and upper side of the box, respectively), the median (middle line) and the minimum to the maximum values (lines extending from box ranges). [file MDC3-10-1620-s006.tif]

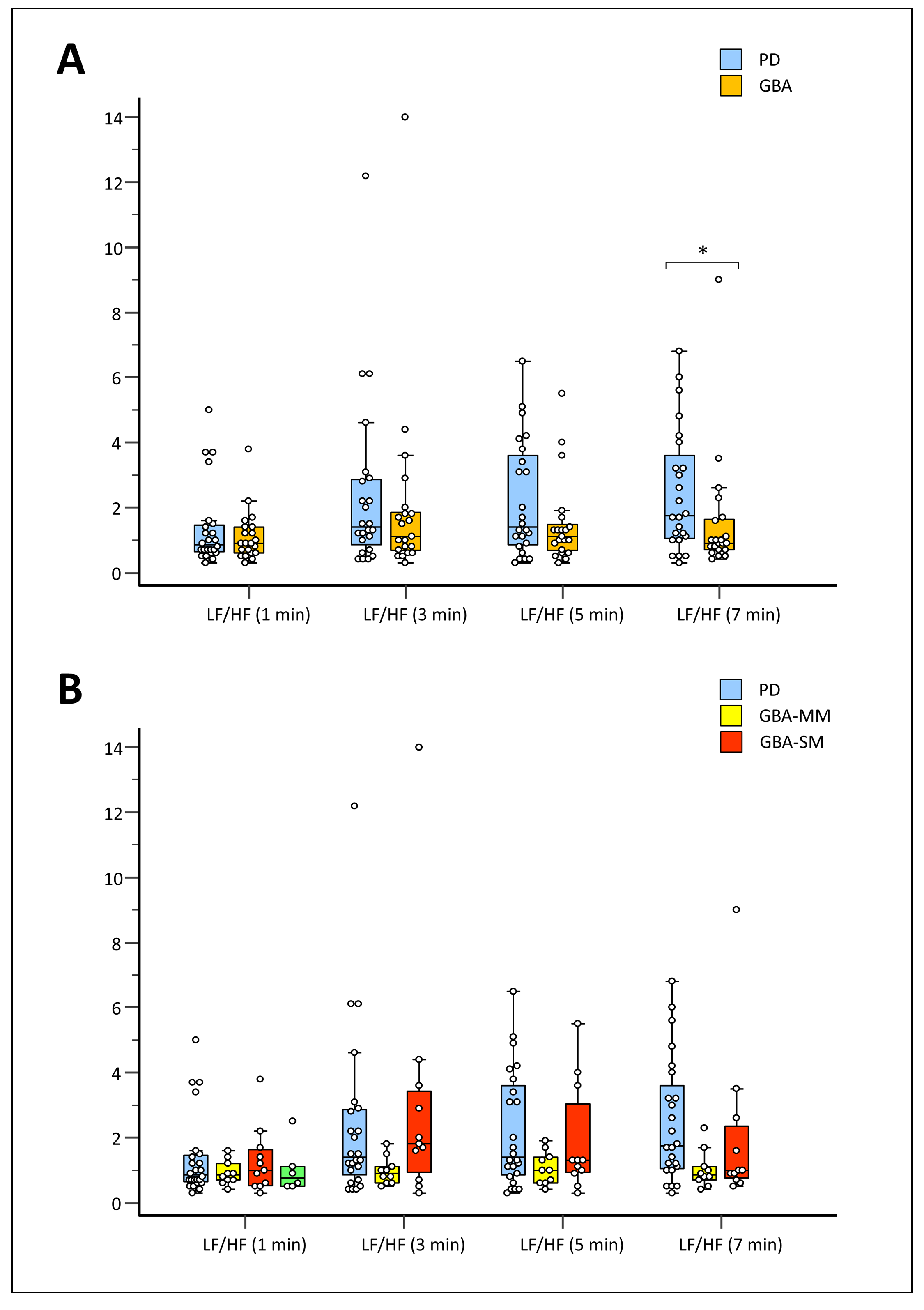

Supplement: Supplementary file 5 — Figure S3. Scatter plots of LF/HF ratio at 1, 3, 5 and 7 min at HUTT for PD noncarriers (PD) and GBA‐carriers (A) and for PD, GBA‐SM, and GBA‐MM (B), showing 25th and 75th percentile (lower and upper side of the boxes, respectively), the median (middle line) and the minimum to the maximum values (lines extending from box ranges), excluding outliers. [file MDC3-10-1620-s004.tif]
