# Supplementary material for: Unraveling Autonomic Dysfunction in GBA‐Related Parkinson's Disease
Source: Mov Disord Clin Pract. 2023 Oct 13;10(11):1620–38. doi: 10.1002/mdc3.13892 (PMC10654845; doi:10.1002/mdc3.13892)
Supplement: Supplementary file 6 — TABLE S1. Instrumental cardiovascular and sudomotor autonomic assessment in GBA‐L444P (GBA‐SM) and GBA‐N370S (GBA‐MM). [file MDC3-10-1620-s005.docx]

| **Supplementary Table 1.** Instrumental cardiovascular and sudomotor autonomic assessment in GBA-L444P (GBA-SM) and GBA-N370S (GBA-MM). | | | | | | | |
| --- | --- | --- | --- | --- | --- | --- | --- |
| **Cardiovascular parameters§** | **GBA-carriers**  **(N=13)** | **Non-carriers**  **(N=13)** | **P-value^a^** | **GBA-L444P**  **(N=7)** | **GBA-N370S**  **(N=6)** | **P-value^b^** | **P-value^c^** |
| **Basal parameters** | | | | | | | |
| Basal RR interval variation | 12.7±6.8 | 15.4±10.4 | 0.24 | 11.2±3.9 | 14.5±9.3 | 0.10 | 0.22 |
| **Cardiac Parasympathetic parameters** |  |  |  |  |  |  |  |
| E:I ratio | 1.2±0.2 | 1.3±0.2 | 0.085 | 1.2±0.1 | 1.3±0.2 | **0.014** | 0.49 |
| %∆E-I (DB vs basal) (%) | 76.9±97.2 | 90.3±121.2 | 0.38 | 53.5±52.4 | 104.3±133.1 | 0.14 | 0.36 |
| DB RR interval variation | 25.7±15.5 | 32.4±20.9 | 0.22 | 21.6±6.8 | 30.6±21.6 | **0.028** | 0.36 |
| Valsalva Ratio (VR) | 1.4±0.2 | 1.7±0.4 | **0.006** | 1.4±0.2 | 1.3±0.1 | **0.019** | 0.11 |
| **Cardiovascular Sympathetic parameters** |  |  |  |  |  |  |  |
| **Valsalva Maneuver*** |  |  |  |  |  |  |  |
| SI 1 (Fall during phase 2) | -20.8±17.4 | -11.8±13.2 | **0.038** | -21.7±20.4 | -19.8±15.4 | 0.058 | 0.23 |
| SI 2 BP Recovery late Phase II | 5.2±25.5 | -3.8±19.3 | 0.18 | 10.9±32.6 | -1.7±13.7 | 0.19 | 0.38 |
| Novak Index (SI3) | -20.3±11.7 | -16.4±14.9 | 0.089 | -18.3±14.2 | -22.6±8.8 | 0.084 | 0.37 |
| Magnitude of phase 4 (SI4) | 5.6±9.4 | 14.3±12.1 | **0.036** | 6.5±9.1 | 4.5±10.7 | 0.13 | 0.11 |
| Pressure recovery time (SI5) | 17.8±15.8 | 11.8±9.2 | 0.20 | 22.8±20.2 | 11.8±5.3 | 0.14 | 0.18 |
| BRSa (SI6) | -1.7±1.7 | -1.8±2.4 | 0.21 | -1.1±1.1 | -2.4±2 | 0.45 | 0.16 |
| **Head-Up Tilt Test (HUTT)**** |  |  |  |  |  |  |  |
| ∆sBP (1 min), mmHg | 7.5±12.6 | 11.9±9.4 | 0.21 | 7±11.9 | 8±14.5 | 0.20 | 0.46 |
| ∆dBP (1 min), mmHg | 17±6.5 | 17.2±8.2 | 0.47 | 16.3±8 | 17.8±4.8 | 0.25 | **0.057** |
| ∆HR (1 min), bpm | 12.5±6.8 | 10.5±6.8 | 0.22 | 13.6±6.8 | 11.2±7.3 | 0.48 | 0.17 |
| ∆sBP (3 min), mmHg | 7.3±12.2 | 6.2±7.5 | 0.40 | 10.1±11.9 | 4±12.8 | 0.25 | 0.32 |
| ∆dBP (3 min), mmHg | 11.1±10.3 | 9.2±7.6 | 0.30 | 14.1±9.7 | 9.7±7.5 | 0.24 | 0.49 |
| ∆HR (3 min), bpm | 14.6±6.5 | 12.5±7.4 | 0.17 | 17.7±6.5 | 11±4.5 | 0.16 | 0.37 |
| ∆HR/∆sBP (3 min), bpm/mmHg | -0.1±3.1 | 1.7±4.9 | 0.14 | -0.9±3.7 | 1±2.2 | 0.18 | 0.30 |
| ∆sBP (5 min), mmHg | -1.3±11.3 | 4.4±8.8 | 0.10 | -3.5±12.8 | 0.8±10.3 | 0.11 | 0.37 |
| ∆dBP (5 min), mmHg | 1.2±8.4 | 6.8±8.9 | **0.083** | 1.3±11.2 | 1±5.5 | **0.091** | 0.29 |
| ∆HR (5 min), bpm | 12.5±8.2 | 12.4±8.9 | 0.48 | 15.5±7.3 | 9.5±8.5 | 0.46 | 0.43 |
| ∆sBP (7 min), mmHg | 0.8±7.8 | 2±8.6 | 0.32 | -2.3±7.3 | 3.8±7.5 | 0.27 | 0.47 |
| ∆dBP (7 min), mmHg | 3.2±5.4 | 3.8±8.3 | 0.41 | 1.8±4.8 | 4.5±6.1 | 0.35 | 0.48 |
| ∆HR (7 min), bpm | 14.8±11 | 10.7±6.6 | 0.14 | 15±7.2 | 14.7±14.7 | 0.19 | 0.26 |
| ∆sBP (10 min), mmHg | -3.2±8.8 | 1.4±10.1 | **0.092** | -1±7.8 | -5.3±9.9 | 0.39 | **0.071** |
| ∆dBP (10 min), mmHg | -0.1±5 | 3.1±10.7 | 0.18 | 1.2±4.4 | -1.3±5.6 | 0.45 | **0.095** |
| ∆HR (10 min), bpm | 13.8±8.4 | 11.7±7.1 | 0.25 | 14.2±8.5 | 13.5±9.1 | 0.41 | 0.15 |
| ∆sBP (1 min post Tilt-down), mmHg | -1.2±18.2 | -5.3±21.6 | 0.31 | 1±13.3 | -3.7±23.8 | 0.36 | 0.39 |
| ∆dBP (1 min post Tilt-down), mmHg | -10.4±12.4 | -19±11.5 | **0.060** | -6.4±10.9 | -15±13.4 | **0.057** | 0.43 |
| ∆HR (1 min post Tilt-down), bpm | 2.7±8.2 | -1.1±6.3 | 0.12 | 0.3±4.6 | 5.5±10.9 | 0.42 | 0.056 |
| **Muscular Sympathetic parameters** |  |  |  |  |  |  |  |
| **Handgrip Test** |  |  |  |  |  |  |  |
| ∆dBP (3 min), mmHg | 2.3±9.8 | 4.6±7.2 | 0.21 | 1.4±5.9 | 3.3±13.6 | 0.051 | 0.18 |
| %∆dBP (3 min vs basal) (%) | 3.3±11.8 | 6±8.9 | 0.23 | 1.6±7.4 | 5.4±16.2 | 0.054 | 0.12 |
| **Spectral Analysis** |  |  |  |  |  |  |  |
| **Frequency-Domain Analysis** |  |  |  |  |  |  |  |
| Basal LF/HF supine | 0.9±0.7 | 0.8±0.7 | 0.33 | 1±1 | 0.9±0.2 | 0.40 | 0.34 |
| LF/HF (DB) | 3.1±4 | 3.7±2.7 | 0.31 | 4.3±5.2 | 1.7±0.9 | 0.44 | 0.10 |
| ∆%(LF/HF-DB vs LF/HF basal) | 262.4±297.3 | 462.1±448.7 | 0.10 | 405.1±350.2 | 96±56.9 | 0.36 | **0.088** |
| LF/HF (1 min) | 1.1±0.9 | 1.3±1 | 0.28 | 1.3±1.3 | 0.8±0.2 | 0.34 | 0.14 |
| LF/HF (3 min) | 2.8±6.3 | 2.8±3.3 | 0.50 | 4.5±8.5 | 0.8±0.2 | 0.31 | 0.12 |
| LF/HF (5 min) | 1.4±1.4 | 2.7±2.1 | **0.002** | 1.6±1.9 | 1.1±0.4 | **0.033** | 0.18 |
| LF/HF (7 min) | 1.5±2.4 | 2.8±2.2 | 0.14 | 2.1±3.4 | 0.9±0.2 | 0.29 | 0.15 |
| LF/HF (10 min) | 4.6±12.4 | 5.2±9.3 | 0.31 | 8.1±17.6 | 1.2±0.7 | 0.23 | 0.11 |
| LF/HF (1 min post-Tilt down) | 1.3±1.7 | 1.1±0.9 | 0.30 | 1.6±2.3 | 1.0±0.6 | 0.24 | 0.34 |
| **Time-Domain Analysis** |  |  |  |  |  |  |  |
| SDNN (ms) | 71.3±7.2 | 119.2±19 | **<0.0001** | 66.9±6.6 | 76.5±3.4 | **<0.0001** | **0.001** |
| SDANN (ms) | 30.7±5.7 | 105.5±17.4 | **<0.0001** | 28.3±2.7 | 33.4±7.2 | **<0.0001** | **<0.0001** |
| SDNN Index (ms) | 21.9±4.4 | 36.4±6.4 | **<0.0001** | 19.4±3.5 | 24.8±3.4 | **<0.0001** | **0.041** |
| r-MSSD (ms) | 10.6±2.7 | 16±2 | **<0.0001** | 8.9±2.4 | 12.5±1.6 | **0.001** | **0.004** |
| pNN50 (%) | 0.7±0.3 | 1.5±0.3 | **<0.0001** | 0.6±0.1 | 0.8±0.4 | **<0.0001** | **0.002** |
| **Sudomotor autonomic parameters** |  |  |  |  |  |  |  |
| **Dynamic Sweat Test (DST)** |  |  |  |  |  |  |  |
| SWEAT /Cm2/MIN (more affected side) | 7.2 [1.7-672] | 8.6 [2.3-41.4] | 0.17 | 4.5 [1.7-672] | 8.5 [3.8-36.7] | 0.19 | 0.35 |
| SWEAT /Cm2/MIN (less affected side) | 15.4 [1.4-74.2] | 10.9 [0-1429] | 0.16 | 22.6 [6.2-74.2] | 14.5 [1.4-18.1] | 0.19 | 0.23 |

**Abbreviations:** PD: Parkinson’s Disease; DLB: Dementia with Lewy Bodies; PKS: parkinsonism; GBA: glucocerebrosidase; SM: severe mutations; MM: mild mutations; E:I (Expiration:Inspiration); DB: Deep Breathing; VR: Valsalva Ratio; SI: Sympathetic Index; BRSa: Baroreflex Sensitivity Index-adrenergic; D: delta; sBP: Systolic blood pressure; dBP: Dyastolic blood pressure; HR: Heart Rate; LF/HF: Low Frequency/High Frequency; SDNN: Standard deviation of NN intervals; SDANN: Standard deviation of the average NN intervals; r-MSSD: Root mean square of successive RR interval differences; pNN50: Percentage of successive RR intervals that differ by more than 50 ms

^§^All parameter are reported as mean±SD, unless otherwise specified. All P-values are calculated with a single tail T test for paired samples. Patients are matched with a 1 to 1 criteria according to sex, age at assessment (±3 yrs) and disease duration (±3 yrs).

^a^ Non-carriers versus matched GBA-carriers. Significant values (p < 0.05) are shown in **bold**

^b^ GBA-SM versus matched Non-carriers. Significant values (p < 0.05) are shown in **bold**

^c^ GBA-MM versus matched Non-carriers. Significant values (p < 0.05) are shown in **bold**

^*^ Cardiovascular Sympathetic parameters during Valsalva maneuver are calculated according to [Novak et al., 2011]. In particular SI 1 (Fall during phase 2) is calculated as percentage variation between baseline mean BP and minimal pressure in phase II; SI 2 BP Recovery late Phase II is calculated as percentage variation between BP at phase IIb and minimal BP in phase IIa; Novak Index (SI3) derives from percentage difference between mean BP at baseline and at phase IIb; Magnitude of phase 4 (SI4) is calculated as variation between maximum value of mean BP during overshoot and baseline mean BP; Pressure recovery time (SI5) corresponds to Time interval between BP drop in phase III and return to BP baseline levels and BRSa (SI6) is calculated as (SI1 + 0.7* SI2)/ PRT

^**^ ∆ of sBP, dBP and HR at each time point of HUTT are calculated with reference to basal sBP, dBP and HR, respectively.

^ⴕ^ According to age-related normative values.
